# CalliFACS: The common marmoset Facial Action Coding System

**DOI:** 10.1371/journal.pone.0266442

**Published:** 2022-05-17

**Authors:** Catia Correia-Caeiro, Anne Burrows, Duncan Andrew Wilson, Abdelhady Abdelrahman, Takako Miyabe-Nishiwaki

**Affiliations:** 1 Primate Research Institute, Kyoto University, Inuyama, Japan; 2 Department of Physical Therapy, Duquesne University, Pittsburgh, Pennsylvania, United States of America; 3 Department of Anthropology, University of Pittsburgh, Pittsburgh, Pennsylvania, United States of America; 4 Graduate School of Letters, Kyoto University, Kyoto, Japan; 5 School of Health and Life Sciences, Glasgow Caledonian University, Glasgow, United Kingdom; University of York, UNITED KINGDOM

## Abstract

Facial expressions are subtle cues, central for communication and conveying emotions in mammals. Traditionally, facial expressions have been classified as a whole (e.g. happy, angry, bared-teeth), due to automatic face processing in the human brain, i.e., humans categorise emotions globally, but are not aware of subtle or isolated cues such as an eyebrow raise. Moreover, the same facial configuration (e.g. lip corners pulled backwards exposing teeth) can convey widely different information depending on the species (e.g. humans: happiness; chimpanzees: fear). The Facial Action Coding System (FACS) is considered the gold standard for investigating human facial behaviour and avoids subjective interpretations of meaning by objectively measuring independent movements linked to facial muscles, called Action Units (AUs). Following a similar methodology, we developed the CalliFACS for the common marmoset. First, we determined the facial muscular plan of the common marmoset by examining dissections from the literature. Second, we recorded common marmosets in a variety of contexts (e.g. grooming, feeding, play, human interaction, veterinary procedures), and selected clips from online databases (e.g. YouTube) to identify their facial movements. Individual facial movements were classified according to appearance changes produced by the corresponding underlying musculature. A diverse repertoire of 33 facial movements was identified in the common marmoset (15 Action Units, 15 Action Descriptors and 3 Ear Action Descriptors). Although we observed a reduced range of facial movement when compared to the HumanFACS, the common marmoset’s range of facial movements was larger than predicted according to their socio-ecology and facial morphology, which indicates their importance for social interactions. CalliFACS is a scientific tool to measure facial movements, and thus, allows us to better understand the common marmoset’s expressions and communication. As common marmosets have become increasingly popular laboratory animal models, from neuroscience to cognition, CalliFACS can be used as an important tool to evaluate their welfare, particularly in captivity.

## Introduction

### The human Facial Action Coding System

The Facial Action Coding System (FACS) was first described by Hjortsjö [1] and later extended by Ekman and colleagues [2–4]. These researchers created a standardised coding system, which identifies and describes in detail the transient facial movements of humans based on their underlying musculature. Whenever a facial muscle contracts, portions of the skin are moved, producing a set of visible appearance changes in the face. In turn, these appearance changes act as cues to identify a specific movement. These facial movements are produced by mimetic muscles and are designated by Action Units (AU). AUs are assigned a distinct numerical code and a descriptive name. For instance, "AU1" corresponds to "Action Unit 1—Inner Brow Raiser" and is coded when the medial portion of the frontalis muscle on the human forehead raises the inner portion of the eyebrows.

It is important to note that low intensity muscular activity (measured for example with Electromyography (EMG) that records electrical activity in skeletal muscles) does not always produce visible appearance changes, or it can generate very minor changes that cannot be reliably identified by visual inspection only. This type of low intensity muscular activity cannot be coded by FACS, as its focus is exclusively on measuring visible facial movements. Hence, minimum criteria are set for each AU, in which the presence of specific visible appearance change(s) are a condition required to code an AU. Nevertheless, studies have demonstrated that higher intensity facial muscle contraction causes visible facial movement, not only in humans but also in other primates [5, 6].

In order to code broad movements or non-mimetic muscle actions, the human FACS manual also includes Action Descriptors (ADs), for example, tongue movements, since these can impact the appearance changes of AUs. The information for all AUs and ADs is compiled in the FACS manual (www.paulekman.com), a self-instructional tool which teaches the user how to identify and code independent movements in the human face.

With FACS, it is possible to systematically and objectively identify facial movements based exclusively on appearance changes that become visible on the face when an underlying muscle contracts. Hence, FACS classifies subunits of movement (i.e. AUs) instead of holistic facial expressions (which are typically composed of more than one AU [7]). This system avoids the subjective interpretation of what observers perceive when seeing a face, and thus, any emotional context biases. FACS is a robust system and accounts for individual differences in facial morphology (for instance, variation in bone structure, fatty deposits, or permanent wrinkles) by using common facial landmarks among individuals and by establishing minimum criteria needed to code an AU. The presence of a neutral face for each individual is thus usually essential for FACS coding, in order to identify the features that may vary between individuals and avoid coding false appearance changes. This is particularly important when coding pictures using FACS, as no dynamic information of the face is present, while in videos it is usually easier to identify a neutral face. For example, some humans may have lip corners slightly turned downwards in a neutral face. In the absence of video which contains important dynamic information, a coder might mistakenly identify the downturned lip corners as a false appearance change of AU15—Lip corner depressor, that as the name implies, pulls the lip corners downwards.

Following the same approach as the human system, FACS has been modified for use with several other primate species: chimpanzees (ChimpFACS [8]), rhesus [9], Barbary [10] and Japanese macaques [11] (MaqFACS), hylobatids (GibbonFACS [12]), and orangutans (OrangFACS [13]), and three domesticated species: dogs (DogFACS [14]), horses (EquiFACS [15]), and cats (CatFACS [16]). The adaptation of FACS for other species is based on the examination of anatomical homologies (e.g. [17–19]) while accounting for species differences in facial morphology. Each of these animal FACS manuals can be accessed online (www.animalFACS.com). The development of animal FACS for different species not only allows new insights into the objective and standardised study of animal communication within each species, but also creates a framework for inter-specific comparative and evolutionary perspectives on facial communication and emotional processes [20, 21]. Hence, FACS are important tools that can be directly applied in the investigation of complex facial displays in humans and other mammals, to not only precisely measure its subunits (i.e. AUs) and tease apart their meaning (e.g. whether certain movements are linked to communication and/or emotion), but also to carry out comparative studies between species, that may be more (e.g. chimpanzees) or less (e.g. dogs) phylogenetically related to humans and ascertain how evolutionary processes drove facial behaviour (e.g. where do human’s highly complex facial expressions come from or which species share which facial displays with humans).

### Why adapt FACS for the common marmoset?

The common marmoset (*Callithrix jacchus*) is a small arboreal primate endemic to Brazil, which has colonised a wide diversity of habitats [22]. Their diet consists primarily of plant exudates, which individuals extract by widely opening their jaws and gouging on tree barks [23]. Common marmosets live in cooperative social groups, and like humans, they usually form bonded-pairs for breeding [24, 25]. These socio-ecological characteristics produce highly complex social cognitive behaviours, including prosociality [26–28], social learning [29], true imitation [30, 31] and altruistic behaviour [32, 33], among others [22, 34, 35]. From a comparative perspective, common marmosets are unique in that they share a lot of these characteristics with humans, which are not shared with more closely related primates [36, 37]. These shared biological traits extend to neurobiology and have recently made the common marmoset one of the most popular comparative animal models used in neuroscience [25, 38], biomedical [39, 40], and cognitive [35] research.

Given their highly complex social and cognitive skills, the steady increase in the use of common marmosets in the laboratory has led to increased welfare concerns, and consequently the need to develop clear indicators for welfare assessment. The common marmoset has a large repertoire of social behaviours including vocalisations, body postures and facial expressions [41–46] which can be used to assess their welfare. In particular, facial expressions are well suited as welfare indicators [47, 48], as they can be correlated not only with communicative behaviours but may also represent underlying emotional states. Although marmosets have been described as "poker-faced" due to their small size and facial colouration [43, 49, 50], a limited number of studies have described facial expressions in the common marmoset (e.g. [41–43]). While some authors have predicted low facial mobility in marmosets when taking into account their socio-ecological characteristics [50, 51] as well as their primitive musculature [52], other researchers [41] reported a surprisingly large number of facial expressions. Kemp & Kaplan [41] compiled 32 facial expressions both from previous published work and from their own experiments, in which common marmosets make use of all facial features, including tufts and tongue movements. This wide range of facial expressions seemed to be displayed in social contexts (e.g. grooming), but also in response to multimodal stimuli (i.e. visual and auditory), suggesting multiple functions, such as communication or emotion expression [41]. Additionally, individuals were found to react differently to positive and negative observed facial expressions, suggesting a role for common marmoset facial expressions as social referencing mechanisms [41]. However, these studies describe facial expressions in common marmosets in a holistic way (i.e. one label for the full facial display that might include several facial movements). Furthermore, some of the facial expressions are identified by broadly descriptive terms (e.g. "slit stare"), while others are identified by emotionally-loaded terms (e.g. "frown"), which make objective comparisons between studies difficult. This is a similar issue to the common research practice of classifying "smiling human faces" as "happy faces". This approach has a number of problems, which include using a subjective and emotionally-loaded label (i.e. not all smiles indicate happiness or even an emotion [53], but smiles tend to be conflated with happiness), and making comparisons between studies difficult (as there are many variations of "happy faces"). While this pioneering work on the common marmoset facial expressions [41–43] has helped us to understand their diverse and complex behavioural repertoire, to date, a more detailed examination of their facial expressions has yet to be undertaken. Therefore, developing a FACS for common marmosets will facilitate the objective study of their facial expressions by avoiding terms that may be hard to be agreed upon by researchers (e.g. "slit stare") or biased by anthropomorphic terms and/or emotional labels (e.g. "frown"). Additionally, due to the high complexity and multi-factorial nature of welfare assessments, FACS can highlight facial movements in common marmosets as an important additional welfare indicator (with potential for both positive and negative indicators), particularly if used in conjunction with other known welfare indicators for this species (e.g. ultrasonic vocalisations [54], cortisol [55], activity patterns and scent markings [56]).

Here, we present the development of an observational tool to measure facial movements in the common marmoset: the CalliFACS (Common marmoset Facial Action Coding System). The CalliFACS is the first objective, systematic and quantifiable tool that will allow users to scientifically measure common marmoset facial movements. It is based on the underlying musculature of the marmoset face and muscular homologies with the human face, and follows the methodology from the human FACS developed originally to study human facial behaviour [2–4]. The aims of this work were: 1) to report the facial musculature and corresponding facial movements of common marmosets through categorisation into AUs, 2) to develop the CalliFACS manual to allow users to become certified in identifying AUs in the common marmoset, and 3) to serve as a reference for coding AUs in future research with common marmosets.

## Methodology

This work follows the Guideline of Care and Use of Nonhuman Primates from the Kyoto University, Primate Research Institute (KUPRI), and was approved by the Animal Welfare and Care Committee of KUPRI (2019–165 and 2020‐064). All work undertaken for this manuscript was purely observational.

Following a similar methodology used in previous FACS adaptations, we employed a three-step methodology to develop the CalliFACS: the first step consisted of determining the facial muscular plan of the common marmoset; in the second step we analysed videos of spontaneous behaviour of marmosets to identify facial movements; finally, in the third step, we combined the anatomical information with the observed facial movements to classify each of these facial movements into Action Units (AUs). Additional movements produced by non-mimetic muscles (e.g. tongue movements) were also classified into Action Descriptors (ADs), and ear movements into Ear Action Descriptors (EADs). This three-step methodology is described in the following sections in detail.

### Determination of the facial muscular plan

**Fig 1:**
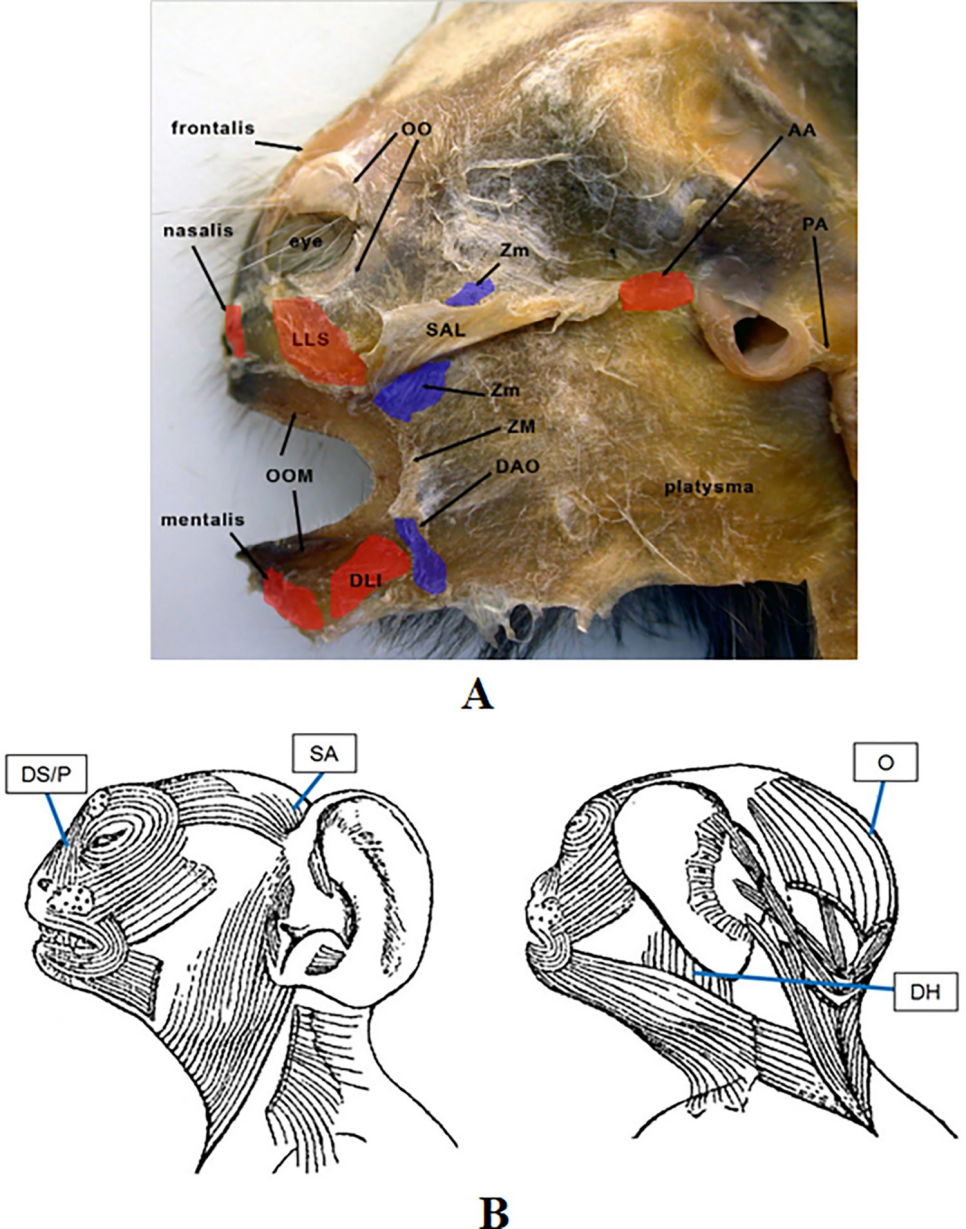
Identification of facial musculature in the common marmoset. **A—**Lateral view of the inverted facial mask dissection technique (from Burrows [51]). **B**—Lateral and dorsal view from dissection diagram (adapted from Huber [52] and Lightoller [57]) illustrating additional muscles not identified in **A**. Labels: **AA**: Anterior auricularis, **PA**: Posterior auricularis, **DAO**: Depressor anguli oris, **DLI**: Depressor labii inferioris, **ZM**: Zygomaticus major, **Zm**: Zygomaticus minor, **SAL**: Superior auriculolabialis, **LLS**: Levator labii superioris, **OO**: Orbicularis oculi, **OOM**: Orbicularis oris, **DS**: Depressor supercilii, **P**: Procerus, **DH**: Depressor helicis, **SA**: Superior Auricularis, **O**: Occipitalis.

**Fig 2:**
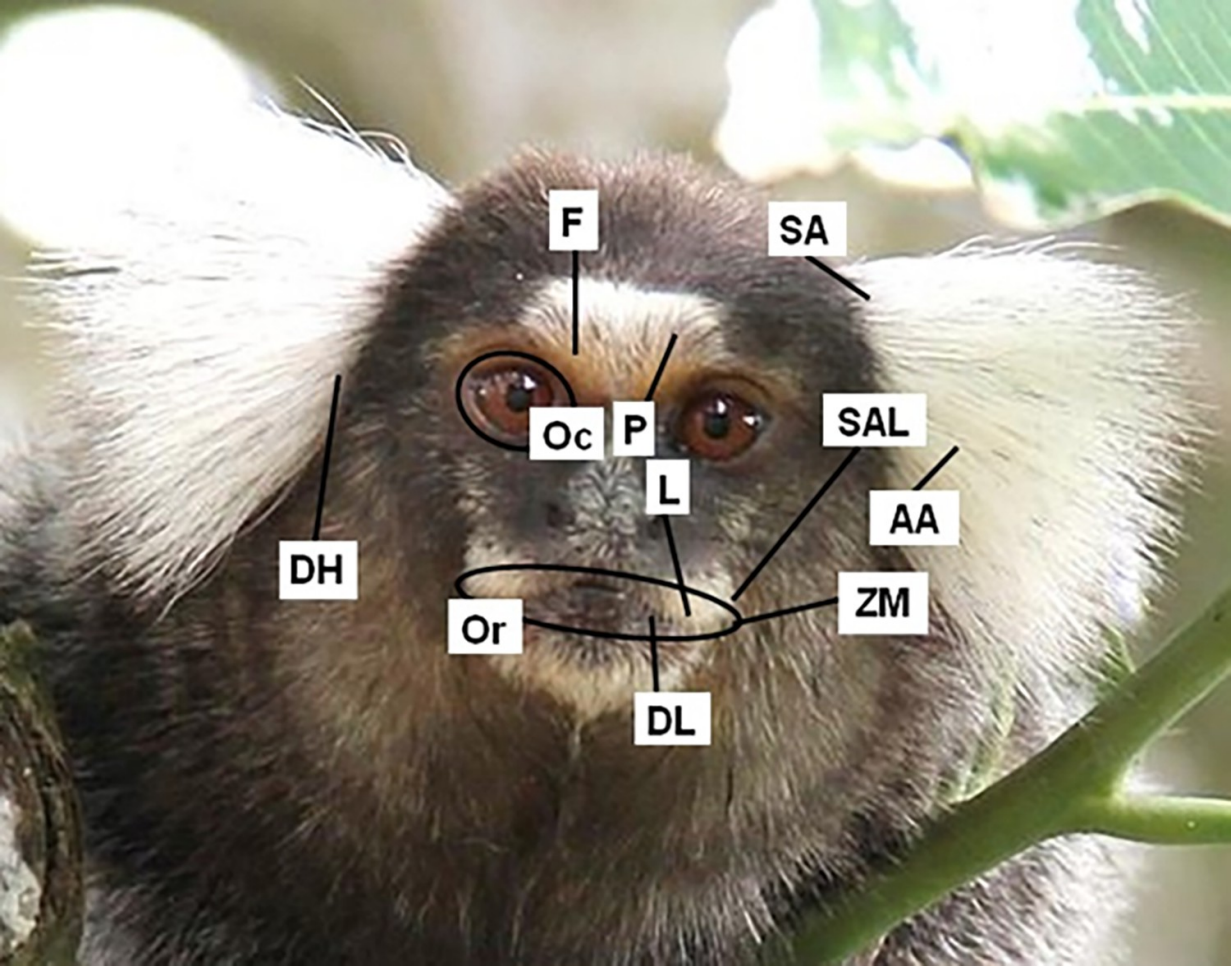
Direction of muscle contraction. Labels represent approximate points of muscle origin (except for the **Or** muscle, which has no clear insertion). Labels: **AA**—anterior auricularis; **DH**—depressor helicis; **DL**—depressor labii inferioris; **F**—frontalis; **L**—levator labii superioris; **Oc**—orbicularis occuli; **Or**—orbicularis oris; **P**—procerus and/or depressor supercilii; **SA**—Superioris auricularis; **SAL**—Superior auriculolabialis; **ZM**—zygomaticus major. The occipitalis (**O**) and the posterior auricularis (**PA**) are not represented here as they are not visible from a frontal view. The **O** inserts in the galea aponeurotica, originates in the occipital region, and contracts ventrocaudally, while the **PA** inserts in the ear cartilage, originates in the occipital region, and contract caudally.

The first step in adapting this system for marmosets was to establish the facial muscular plan from published literature [51, 52, 57] (**Fig 1**) and subsequently compare it with human facial musculature in order to identify possible functional homologies [58] (**Table 1**). The proposed muscle function in common marmosets is illustrated in **Fig 2**, based on points of origin, insertion and fibre direction. Due to ethical concerns [5, 13], and technical limitations (e.g. needle size), the muscle function here described for the common marmoset was based on published dissections [51, 52, 57] and functional homologies [58] with humans and other primates, instead of intramuscular electrical stimulation. For the development of the FACS for humans, chimpanzees [5] and rhesus macaques [6], intramuscular electrical stimulation was performed to confirm and validate facial muscle function. Similarly to other FACS adaptations [13, 16], for CalliFACS this invasive procedure was not carried out since the functional homologies [58] were established and it avoids ethical concerns [20].

**Table 1:** Comparison between FACS Action Units (AU) for humans [3] and common marmosets [51, 52, 57] according to underlying musculature. ✓- present, **x**—absent.

AU code	AU name	Underlying muscle	Human	Common marmoset
**AU1**	**Inner Brow Raiser**	Frontalis (medial)	✓	**x**
**AU2**	**Outer Brow Raiser**	Frontalis (lateral)	✓	**x**
**AU1+2**	**Brow Raiser**	Frontalis^1^	✓	✓
**AU4**	**Brow Lowerer**	Procerus, Depressor supercilii, Corrugator supercilii	✓	**x**
**AU41**	**Glabella Lowerer**	Depressor supercilii^2^ and/or Procerus^3^	**x**	✓
**AU5**	**Upper Lid Raiser**	Orbicularis oculi^1^	✓	**x**
**AU6**	**Cheek Raiser**	Orbicularis oculi^1^, pars orbitalis	✓	✓
**AU7**	**Lid Tightener**	Orbicularis oculi^1^, pars palpebralis	✓	**x**
**AU43**	**Eye closure**		✓	✓
**AU45**	**Blink**		✓	✓
**AD47**	**Half-blink**		**x**	✓
**AU8**	**Lips Towards Each Other**	Orbicularis oris^1^	✓	**x**
**AU9**	**Nose Wrinkler**	Levator labii superioris alaeque nasi	✓	**x**
**AU10**	**Upper Lip Raiser**	Levator labii superioris^1^	✓	**x**
**AU110**		Superior auriculolabialis^1^, Zygomaticus minor	**x**	✓
**AU109+110**	**Nose Wrinkler and Upper Lip Raiser**	Levator labii superioris^1^, Superior auriculolabialis^1^	**x**	✓
**AU11**	**Nasiolabial Furrow Deepener**	Zygomatic minor^1^	✓	**x**
**AU12**	**Lip Corner Puller**	Zygomatic major^1^	✓	✓
**AU13**	**Cheek Puffer**	Caninus (or Levator anguli oris)	✓	**x**
**AU14**	**Dimpler**	Buccinator	✓	**x**
**AU15**	**Lip Corner Depressor**	Depressor anguli oris^1^	✓	**x**
**AU16**	**Lower Lip Depressor**	Depressor labii inferioris^1^	✓	✓
**AU17**	**Chin Raiser**	Mentalis^1^	✓	**x**
**AU18**	**Lip Pucker**	Incisivii labii (superioris and inferioris)	✓	**x**
**AU118**		Orbicularis oris^1^, Buccinator	**x**	✓
**AU20**	**Lip Stretcher**	Risorius	✓	**x**
**AU21**	**Neck Tightener**	Platysma myoides^1^	✓	**x**
**AU22**	**Lip Funneler**	Orbicularis oris^1^	✓	**x**
**AU23**	**Lip Tightener**		✓	**x**
**AU24**	**Lip Pressor**		✓	**x**
**AU25**	**Lips Parted**	Orbicularis oris^1^, Levator labii superioris^1^, Depressor labii inferioris^1^, non-mimetic muscles	✓	✓
**AU26**	**Jaw Drop**		✓	✓
**AU27**	**Mouth Stretch**		✓	✓
**AU28**	**Lip Suck**	Orbicularis oris^1^	✓	**x**
**AU38**	**Nostril Dilator**	Nasalis^1^	✓	✓
**AU39**	**Nostril Compressor**	Nasalis^1^, Depressor septi nasi	✓	**x**

^1^Muscles described in the common marmoset inverted facial mask dissection by Burrows [51].

^2^Described by Huber [52].

^3^Described by Lightoller [57].

### Identification of facial movements

The second step for developing the CalliFACS consisted of watching video recordings of spontaneous facial movements of common marmosets with the aim of 1) identifying the facial movements (AUs, ADs, and EADs) common marmosets can potentially display, 2) finding at least one clear example of each facial movement, and 3) extracting short videos to illustrate these examples (included in this manuscript as **Supporting Information Videos**). A sample of 121 videos (Mean±SD: 164±200 s) were watched (by CCC) frame-by-frame, totalling approximately five hours of videos. The videos focused mostly on the head, and included variable frame rate (30–240 FPS). This sample featured approximately 100 individuals (it was not always possible to verify the identity of the individuals in the footage) in a variety of populations (e.g. captive individuals in zoos, sanctuaries, research facilities, and kept as pets, comprising approximately 4h25m of video, as well as wild and semi-urban individuals comprising approximately 46m of video), and contexts (including potentially positive, negative and neutral contexts: e.g. grooming, feeding, play, human interaction, veterinary procedures).

Part of the footage used to develop CalliFACS (approximately 2h36m) was collected at the Primate Research Institute, Kyoto University (KUPRI), by filming common marmoset spontaneous behaviour in their home enclosures *ad libitum*, using a GoPro Hero7 and GoPro Hero8 cameras. The individuals were housed indoors, in group (W700 x D700 x H1500 mm or W910 x D700 x H1600 mm) or paired cages (W1200 x D600 x H1000 mm), and kept at 28±5°C with a 12h light cycle. Environmental enrichment was provided, such as gum feeders, wooden and metal climbing structures, platforms, swings, hanging/chewing objects, and occasionally novel objects. Water was available *ad libitum*. They were fed on 50ml (20–30g) of pellets (SPS, Oriental Yeast Co. ltd., Tokyo, Japan) twice a day, supplemented with apple and quail’s eggs three times a week, banana twice a week, and occasionally mealworm (the larva of Tenebrionidae). KUPRI care staff monitored the health and welfare of the individuals daily, using criteria such as faecal condition, appetite, hair condition, and movement.

Other videos of common marmosets were reused from other ethically approved research projects and veterinary procedures unrelated to the present work (approximately 1h08m), or sourced from online public databases (e.g. YouTube.com, MarmosetCare.com, all with a Creative Commons Licence or approval from the video owner, approximately 1h23m). Therefore, no negative contexts (e.g. pain, distress) were induced during the current work and/or solely for the purpose of developing CalliFACS. Still images were extracted from videos in some instances or downloaded from public databases (e.g. Pixabay.com, Unsplash.com) to illustrate particular facial features or aid in the identification of appearance changes. This dataset was deemed sufficient for the aim of developing a new CalliFACS, as we successfully identified at least one example of each movement from all facial muscles in the common marmoset (**Table 1**). In line with previous AnimalFACS adaptations [e.g. 14], analysis of videos for a species is conducted only until one or two clear examples of facial movements (in particular AUs) are found for all facial muscles described in the common marmoset. We recognise that it is possible that movements displayed in very specific contexts (e.g. copulation) or that are rare may be missing from our video sample. However, if additional movements are found in the future, they can be added to the CalliFACS through the certification website for AnimalFACS (www.animalFACS.com).

As this video database was not fully or systematically coded for all occurrences of each AU (as this was not needed to identify clear examples of each AU), we did not perform reliability analysis in this step (in line with Human and AnimalFACS previous adaptions).

Although a systematic measurement of AU duration was beyond the scope of the CalliFACS development, it was noted that most marmoset AUs were extremely quick, with some AUs below the lower threshold for human visual awareness (20-40ms [59–61]). As a baseline for comparison, micro-expressions in humans, which are hard to detect without training, are defined as being on average 300ms, and under 500ms [62]. Spontaneous eye blinks have also been reported to occur less frequently and each blink is performed much faster in common marmosets (5.4 blinks per minute, duration of 178.2ms [63]) than in humans (e.g. 20.8 blinks per minute, duration of 403.6 +-52.6ms [63, 64]). Therefore, in order to detect AUs in marmosets it is essential to record high frame rate footage and analyse it frame-by-frame and/or slow-motion.

### Classification of facial movements into action units, action descriptors and ear action descriptors

**Fig 3:**
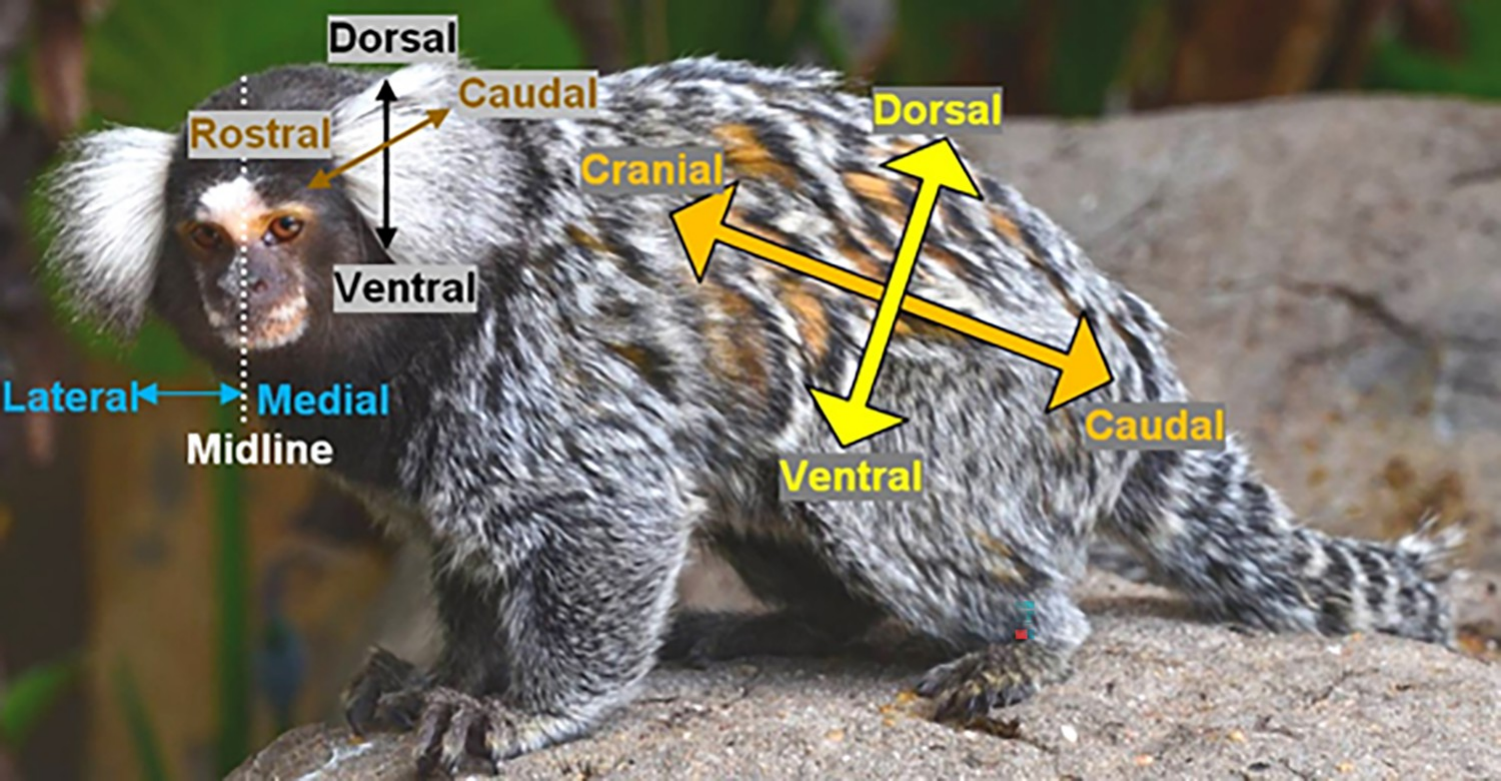
Spatial representation of directional terminology for a quadruped animal. **Cranial**: towards the cranium, along the long axis of the trunk (craniocaudal axis). **Rostral**: towards the apex of the nose, along the long axis of the head (rostrocaudal axis). **Caudal**: towards the tail or back of the head, along the long axis of the trunk or head, respectively. **Dorsal**: towards the spinal column or the top of the head, along the short axis of the trunk or the short axis of the head, respectively (dorsoventral axis). **Ventral**: towards the abdomen or the underside of the head, along the short axis of the trunk or the short axis of the head, respectively. **Medial**: towards the medial plane (represented by the **midline**) of the body or head. **Lateral**: from the medial plane, towards the left or right side of the body or head. These terms can be combined to describe a movement in additional directions, as for example: dorsocaudal, ventrocaudal, dorsocranial, ventrocranial.

In the last step of the CalliFACS development, the anatomical plan and behavioural video analysis were combined to describe the facial movements observed in the videos using specific directional and anatomical terms (**Fig 3**). These were classified according to codes used in previous FACS (including AUs, ADs and EADs), following functional muscular homologies wherever possible, or creating new codes whenever the homologies were not identified (for instance, by adding "1" before the code used for the original FACS). All the AUs found in common marmosets are presented in **Table 1** along with the corresponding muscles. Additionally, **Table 2** describes the ear (EADs), tuft and scalp (ADs) movements, whilst **Table 3** includes other ADs found in the common marmoset.

**Table 2:** Comparison between FACS movements for ears and related movements of the tufts and scalp for humans [3] and common marmosets [51, 52, 57], according to underlying musculature, including Ear Action Descriptors (EAD) and Action Descriptors (AD). ✓- present, x—absent.

EAD/AD code	EAD/AD name	Underlying muscle	Human	Common marmoset
**EAD1**	**Ears Forward**	Anterior auricularis^1^	**x**	✓
**EAD2**	**Ears Elevator**	Superior auricularis^4^	**x**	**x**
**EAD3**	**Ears Flattener**	Posterior auricularis^1^	**x**	✓
**EAD105**	**Ears Downwards**	Depressor helicis^3^	**x**	✓
**AD101**	**Scalp Retraction**	Occipitalis^2^^,^^3^, Posterior auricularis^1^	**x**	✓
**AD300**	**Tufts Upwards**	unknown	**x**	✓
**AD301**	**Tufts Downwards**	unknown	**x**	✓

^1^Muscles described in the common marmoset inverted facial mask dissection by Burrows [51].

^2^Described by Huber [52].

^3^Described by Lightoller [57].

^4^Described by Lightoller [57] as a weak muscle originating from the galea and inserting into the auricular cartilage.

**Table 3:** Comparison between other FACS Action Descriptors (AD) for humans [3] and common marmosets. ✓- present, x—absent.

AD code	AD name	Human	Common marmoset
**AD181**	**Lip Smacking**	**x**	✓
**AD19**	**Tongue Show**	✓	✓
**AD190**	**Tongue Downwards**	**x**	✓
**AD191**	**Tongue Curl**	**x**	✓
**AD119**	**Lick**	✓	✓
**AD29**	**Jaw Thrust**	✓	✓
**AD30**	**Jaw Sideways**	✓	✓
**AD31**	**Jaw Clencher**	✓	**x**
**AD32**	**Bite**	✓	**x**
**AD33**	**Blow**	✓	**x**
**AD34**	**Puff**	✓	**x**
**AD35**	**Suck**	✓	**x**
**AD36**	**Bulge**	✓	**x**
**AD37**	**Lip Wipe**	✓	**x**
**AD40**	**Sniff**	✓	✓
**AD50**	**Vocalisations**	✓	✓
**AD80**	**Swallow**	✓	✓
**AD81**	**Chewing**	✓	✓
**AD160**	**Body Shake**	**x**	✓

### Facial morphology in the common marmoset

When identifying facial movements in a new species, it is important to consider, study and familiarise oneself with the facial morphology, particularly in the aspects that differ from the human face (as presumably, expertise and familiarity with human faces is common for all humans). As facial morphology is very unique to each species, so are facial landmarks and other anatomical reference points important to identify facial movements. Hence, next is a description of the common marmoset facial morphology.

**Fig 4:**
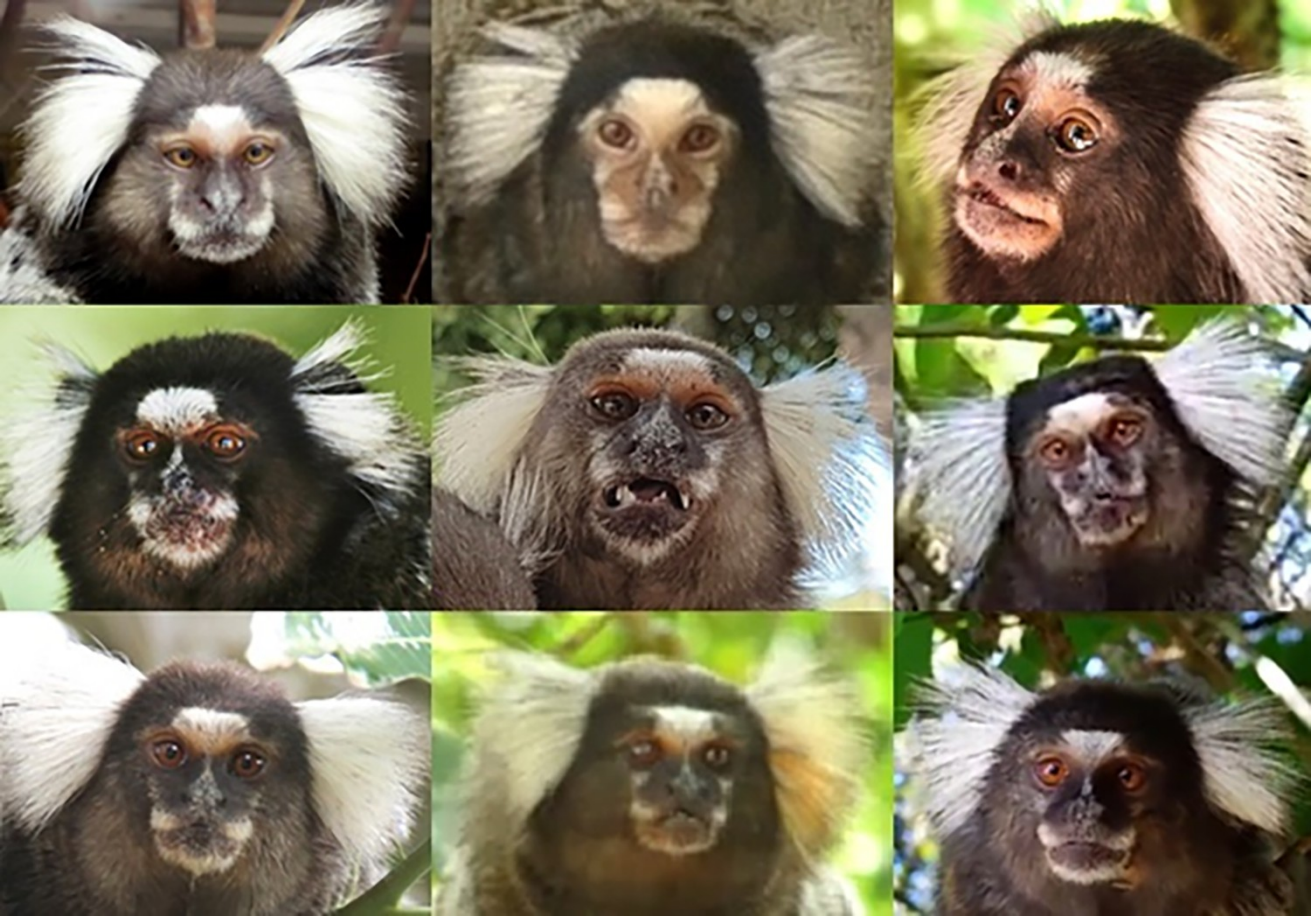
Variation of facial coloration in wild and captive marmosets.

The common marmoset adult skull measures approximately 45 mm in length and 29 mm in width [65], which makes their faces particularly small in comparison to other primate species. The neck area is not well defined. Although there might be some variation in facial coloration depending on population/individual differences (**Fig 4**), in general, the facial area of marmosets is covered in short cream-colored hairs, contrasting with the surrounding longer and darker hair. There is usually variation in contrasting dark and light patches of skin and hair across the face and often a darker patch in the mid-face area. All common marmosets display a conspicuous patch of white/cream skin on the glabella region covered by white hair. This white patch varies in size and its size seems to be dependent on vocal communication development in common marmosets [66]. They also have large ears (the pinna is approximately 2cm high and 1.5cm wide) relative to head size, that in frontal view are usually covered with the characteristic white tufts. The oval nasal openings are rostrolaterally oriented with a relatively large internarial distance. The mouth can be opened widely, with a relatively large mandible and a prominent mandibular angle [65].

**Fig 5:**
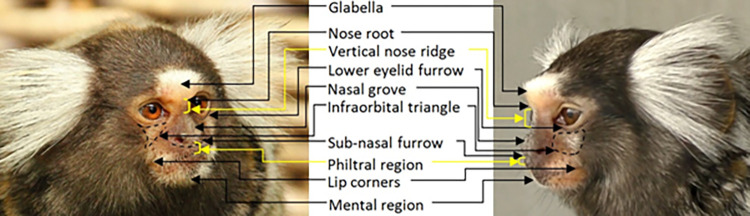
Facial landmarks in common marmosets.

**Fig 6:**
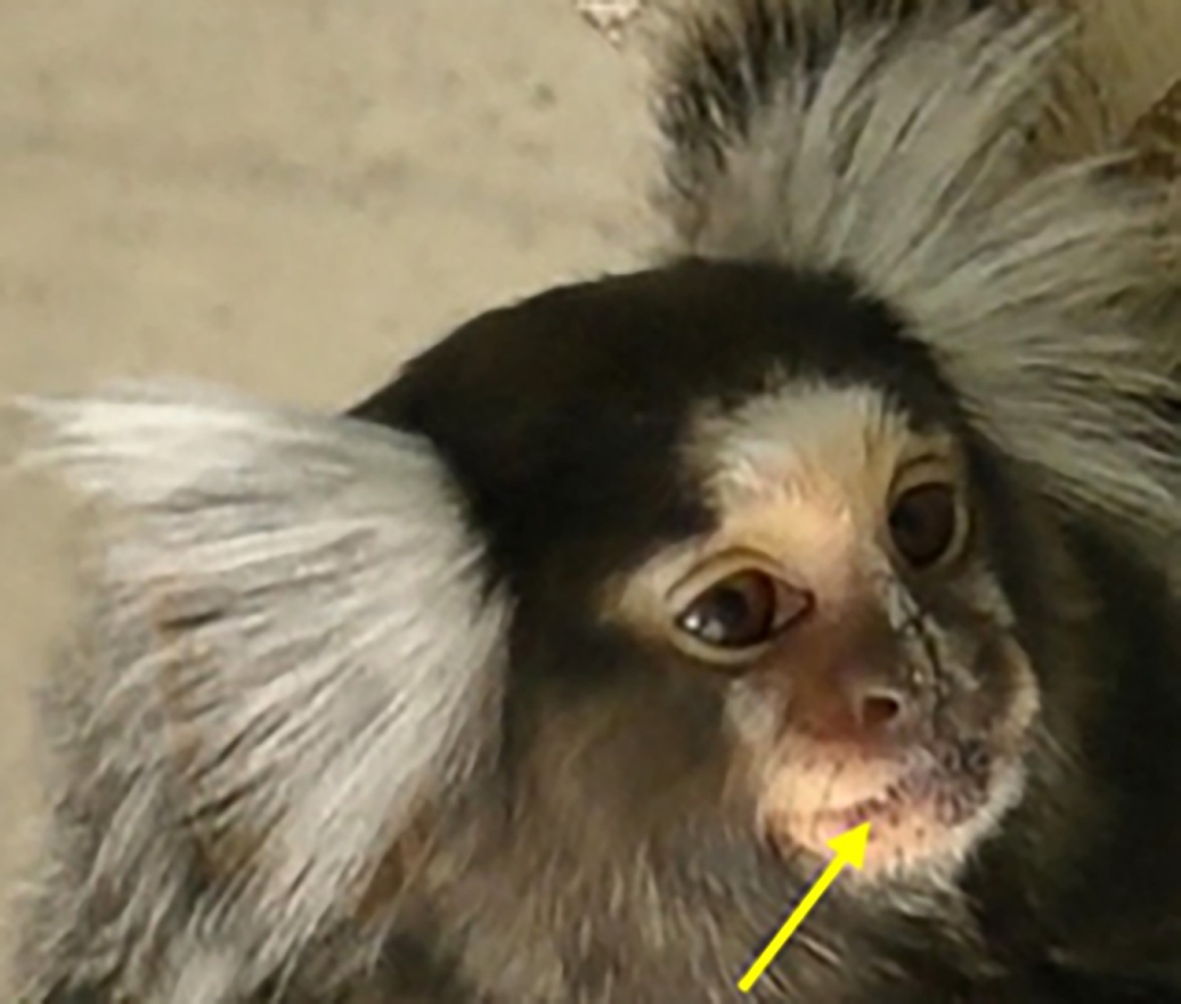
Variation in teeth visibility. One of the upper tooth tips is visibly protruding from the lips, indicated by the yellow arrow.

Regarding their facial landmarks (**Fig 5**), marmosets present a more or less prominent browridge with a slightly more salient glabella (compared to the lateral browridge portions). The infraorbital furrow is hardly indistinguishable from the infraorbital triangle, and unlike in macaques and humans, the bulge immediately under the lower eyelid furrow is absent. The nasal furrow and philtral region seem to form a continuous depression in some individuals. The upper lip is not straight, but medially curved upwards, acting as a false indicator as if the medial portion of the upper lip is pulled towards the nose. Another false indicator to note in some individuals relates to the arrangement of the teeth (**Fig 6**), where some of the teeth might protrude from the lips even when the mouth is closed. Seen from a frontal view, the mouth has an inverted "V" shape.

### How to use CalliFACS as a coding tool

In **Fig 1**, a diagram of the muscular plan of the common marmoset shows where the muscles are located on their face. In **Fig 2**, the direction of action is presented for each muscle. The label with the muscle abbreviation marks the approximate location of the muscle origin on the bony structure. The opposite end of the line indicates approximately where the muscle attaches to the face skin (also known as its insertion). When a muscle contracts, it pulls the skin towards the label position, usually bunching or wrinkling the skin perpendicularly to the direction of muscle pull. For example, in AU1+2 the frontalis muscle originates from the frontal bone and attaches at the glabella region [51]. These two diagrams illustrate the underlying musculature and its function, which help in understanding the facial appearance changes produced during AUs. For specific anatomical terms and definitions, please see the glossary in **S1 Text**.

This manuscript includes all the AUs, ADs and EADs found in the common marmoset, along with a list of appearance changes describing in detail the visual results of each muscular movement on the face of the common marmoset. Each movement is illustrated by still images and/or short video-clips (most accompanied by a slow-motion version, see **Supporting Information Videos**). Throughout the text, AU, AD and EAD codes will be used frequently with their numerical codes only, so please refer to **Tables 1–3** for code identification.

As with all FACS systems, any person interested can become a certified CalliFACS coder, as due to the objective nature of FACS, no experience with FACS or the target species is needed to become a certified coder in a certain species (e.g. [15]). However, to become certified in CalliFACS, it is required to self-study the CalliFACS Manual (i.e. this manuscript and its Supporting Information Videos) before taking a certification test (i.e. the 24 videos described in the next section "Coding reliability") to ensure system reliability. CalliFACS learners should carefully study the anatomical information (see previous sections on anatomy and morphology), the appearance changes, and the minimum criteria here described for each AU/AD/EAD (see results section) and illustrated in the Supporting Information Videos, before taking the certification test. The CalliFACS certification test is freely available upon request at www.animalFACS.com. We suggest the repeated visualisation of the Supporting Information Videos with, for example, the VLC media player.

### Coding reliability

We tested inter-observer reliability between three FACS coders (CCC: certified in HumanFACS [3] and in all the AnimalFACS developed to date [8, 9, 11–16]; DAW: certified in MaqFACS [9] and ChimpFACS [8]; and AA: certified in MaqFACS [9]) by coding 24 short clips (not used to describe the AUs). Inter-observer reliability was used to: (1) confirm all coders could reliably identify AUs included on the CalliFACS manual, and (2) to refine the descriptions of AUs through discussion when agreement between coders on a particular AU was low. This was followed by additional rounds of coding using the same 24 clips to confirm that inter-observer reliability had sufficiently improved. In each coding round, two of the coders (DAW and AA) were blind to each other’s scores and to the third coder (CCC) scores.

The coders’ overall reliability (Wexler’s index [67], Eq (1)) and the AUs independent coding agreement (calculated through the average of each AU agreement) from a first round of coding (**Table 4**) indicated a low overall agreement between coders of 52%. A second round of coding increased the overall reliability to 69% (**Table 4**), although this was not yet considered a "good" agreement [4, 68]. In a third and final round of coding, we obtained a very good mean agreement of 82% (including 89%, 84% and 74% for each pair of coders) from the Wexler’s index [67] (1), and also a good independent coding agreement on most AUs (**Table 4**). The “coding key” together from these reliability clips will act as a certification test for future CalliFACS coders, in which an agreement score of 70% or more will be needed (as per the Wexler’s index and [68]). The “coding key” excluded the occurrences of AUs that did not reach perfect agreement between the three coders in a particular clip. However, each AU is present in more than one clip, so future CalliFACS coders will be tested on all AUs and most ADs.


$${\;Wexler}^{\prime }s\;index=\frac{(Number\;of\;AUs\;on\;which\;coder\;1\;and\;Coder\;2\;agreed)\times 2}{The\;total\;number\;of\;AUs\;scored\;by\;the\;two\;coders}$$ (1)

**Table 4:** Mean Wexler’s index [67] (1) and independent coding agreement for each AU, AD and EAD in the three coding rounds. NA denotes instances where all coders agreed that a particular Action was not present in any of the clips.

	Round 1	Round 2	Round 3
Wexler’s index	0.52	0.69	0.82
AU1+2	0.45	0.57	0.82
AU41	0.23	0.75	0.71
AU6	0.79	0.79	0.79
AU43	0.43	0.60	0.43^1^
AU45	0.63	0.96	0.96
AU47	0.60	0.71	0.71
AU110	0.38	0.40	0.75
AU109+110	0.21	0.38	0.67
AU12	0.89	0.86	0.92
AU16	0.78	0.78	0.89
AU118	0.38	0.50	0.60^1^
AU25	0.88	0.88	0.95
AU26	0.71	0.60	0.90
AU27	0.69	0.86	0.86
AU38	0.50	0.75	0.75
AD181	1.00	1.00	1.00
AD19	0.75	0.75	0.75
AD190	0.75	0.75	1.00
AD191	NA	NA	NA^2^
AD119	NA	NA	NA^2^
AD29	0.50	0.86	0.86
AD30	0.00	0.00	0.00^1^
AD40	NA	NA	NA^2^
AD80	NA	NA	NA^2^
AD81	NA	NA	NA^2^
AD160	NA	NA	NA^2^
AD101	0.82	0.82	0.90
AD300	0.00	0.00	0.00^1^
AD301	0.67	0.67	1.00
EAD1	0.00	0.00	0.00^1^
EAD3	0.43	0.86	0.75
EAD105	NA	NA	NA^2^

^1^Low agreement due to rarely coded AUs/ADs/EADs (<3 occurrences), not due to low agreement between coders.

^2^NAs were only scored for ADs and one EAD105.

## Results

The results here presented are intended not only as a report of the facial movements found when developing the CalliFACS for common marmosets, but also as a manual for future CalliFACS coders to learn to identify these facial movements and a guide for any CalliFACS coding post-certification. Following from the three rounds of coding reliability (described in the previous section), each movement is described in detail and exemplified with pictures and videos in supporting information.

### Action units

We report each AU found in the common marmoset (from here onwards marmoset), with a numerical code, a descriptive name, and a brief comparison of the anatomical features between humans and marmosets, and if relevant, other primates. The following information is then given:

**A. Proposed muscular basis:** Muscle(s) that produces the AU (**Table 1**);

**B. Appearance changes:** List of multiple and redundant cues (e.g. face feature movement of shape change, movement direction, and formation or deepening of wrinkles, in relation to facial landmarks, **Fig 5**) that help to identify when an AU occurs. Video (see **Supporting Information Videos**) and photo examples are also presented illustrating different appearance changes;

**C. Minimum criteria** to code an AU: visible appearance change(s) that when present are sufficient to code an AU;

**D. Subtle differences between AUs:** Wherever necessary, a comparison of similar AUs that can be confused or that share some appearance changes.

#### Upper face action units

*AU1+2—Brow Raiser*. The brow area presents marked differences between humans and marmosets, which affect upper face AUs and respective appearance changes. In humans, the forehead (portion of the frontal bone between the eyebrows and hairline) and the eyebrows (hair strips located on the supraorbital ridges) are morphological and anatomical features unique to humans. The eyebrows are highly visually salient on the naked forehead and are part of important appearance changes for several AUs. Marmosets do not have a forehead or eyebrows, but the area of the head located on the frontal bone is designated as the frontal region and the salient area above the eyes as a browridge. However, the frontal region is not above the supraorbital ridges, but instead sits almost on a transverse plane in relation to the face. The frontal region and browridge are covered in short dark brown/black hair. The browridge is slightly more salient than in humans, particularly on the medial portion of the browridge or glabella. The glabella in marmosets is covered in short white/cream hair in the shape of an inverted triangle, creating a high contrast area with the surrounding dark hair.

In the **human FACS**, AU1 - "Inner brow raiser" raises the medial portion of the eyebrow and AU2 - "Outer brow raiser" raises the lateral section of the eyebrow creating wrinkles on the forehead. These two movements can be coded separately in humans and unilateral movements (i.e. in one hemiface only) are often observed. In **marmosets**, similar to other primates, AU1 and AU2 are not observed independently, and so are only coded in a combined movement noted as AU1+2 (**S1a, S1b, S2a** and **S2b** Videos). AU1+2 is also only observed bilaterally in marmosets, i.e. on both sides of the face. Furthermore, the brow movement in marmosets can be seen along the whole width of the browridge. This is similar to the human brow movement, but unlike other primates (e.g. macaques) where movement is concentrated in the medial portion of the brow.

**A. Proposed muscular basis:** Frontalis.

**B. Appearance changes**:

1. The browridge moves dorsally, rolling over the frontal region.
2. The glabella appears flattened, widened and less salient.
3. The underbrow region is more visible, with skin appearing to stretch and the eye cover fold may be more exposed.
4. In more intense movements, a depression may form between the browridge and the frontal region.
5. In more intense movements, the hair in the frontal region might move caudally.

**C. Minimum criteria:** Dorsal movement of the glabella or browridge.

**D. Subtle differences between AUs:** Head movements (e.g. head up) or changes in camera angle might make the underbrow more visible (appearance change 3). Consequently, it may seem that AU1+2 is acting, but this movement should only be coded if the minimum criterion is present, in which the browridge is seen moving up.

*AU41—Glabella Lowerer*. The glabella area (i.e. space between the brows) also differs considerably between humans and marmosets. While in **humans** the glabella is a flat area of (usually) naked skin between the brows, in **marmosets** the brightly coloured glabella area between the brows protrudes due to a fatty deposit and forms a highly visible inverted triangle shape.

**Fig 7:**
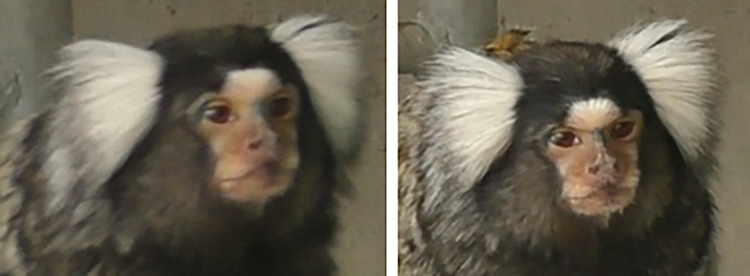
Left: Neutral; Right: AU41—Glabella Lowerer. Still frames from S3 Video.

**Humans** can produce AU4—Brow Lowerer, by the contraction of three different muscles (procerus, depressor supercilii and corrugator). These muscles pull the brow downwards medially and/or laterally, and uniquely to humans, can corrugate the brows, i.e. the brows come closer together and create wrinkles on the glabella. In **marmosets**, AU4 is not observed, as there is no corrugation. The only movement observed is that the brows are pulled down, with the movement more conspicuous in the glabella. Hence, marmosets produce AU41—Glabella Lowerer instead (**Fig 7, S3A, S3B, S4A, S4B, S5A** and **S5B** Videos).

**A. Proposed muscular basis:** Depressor supercilii, procerus.

**B. Appearance changes**:

1. The glabella moves ventrally.
2. The glabella bulges, becoming more conspicuous; its shape might change into a more inverted trapezoid shape (i.e. from ▽ to).
3. The underbrow region is less visible, the eye cover fold may disappear from view and the root of the nose might also be covered.
4. The eyes may become narrower.
5. The face appears de-elongated.
6. In more intense movements, the lateral aspects of the browridge may follow the glabella ventral movement, but it may be harder to detect.
7. In very intense movements, the root of the nose might be completely covered (**S6a** and **S6b** Video).

**C. Minimum criteria:** Ventral movement of the glabella or browridge.

**D. Subtle differences between AUs:** Even though AU1+2 and AU41 act in opposite directions (dorsal and ventral respectively), in **humans** they can be coded simultaneously, impacting each other’s appearance changes and creating new appearance changes. In contrast, in **marmosets** they are mutually exclusive, i.e. they cannot be coded simultaneously, and hence do not share any appearance changes. However, these two movements are sometimes observed in succession. In these cases, the release of AU41 to neutral might be difficult to distinguish from a weak AU1+2, and the release of AU1+2 to neutral might be confused with a weak AU41. In order to define when to code one or the other, comparison with the neutral browridge for each individual might be necessary.

*AU6—Cheek Raiser*. **Human** cheeks are formed by deposits of fat sitting on the zygomatic bone, just below the eyes. The outer layer of the muscle surrounding the eyes (orbicularis occuli pars orbitalis) contracts to pull the surrounding skin towards the eye, decreasing the infraorbital triangle (IOT, **Fig 5**) area and raising the cheeks. In **marmosets**, the inner and outer portions of the orbicularis occuli muscle have been described as having no apparent difference other than the palpebralis part having no bone attachment [57], hence it appears that the concentric eye muscle is less differentiated in this species. Furthermore, there are no evident fat deposits on the zygomatic bone that form the human-like cheeks, so the appearance changes are significantly different from humans, instead affecting the skin around the eye more globally in marmosets. Likely due to these anatomical differences, in humans AU6 can be coded as an independent action, while in marmosets it is mostly observed with AU43/45.

**A. Proposed muscular basis:** Orbicularis occuli (pars orbitalis).

**Fig 8:**
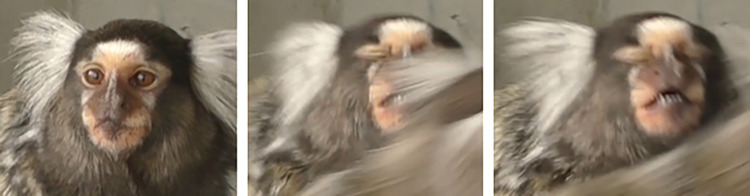
Left: Neutral; Centre: AU6—Cheek Raiser at its apex; Right: AU6—Cheek Raiser returning to neutral. Still frames from S8 Video.

**B. Appearance changes**:

1. The IOT decreases in size and bulges (**Fig 8, S8A** and **S8B** Video).
2. The skin of the IOT is pulled towards the eye.
3. Deepens the infraorbital furrow.
4. Even if eye closure is not present, it narrows eye aperture.
5. When eye closure is present, it can make the eyelids appear compressed and bulging.
6. Wrinkles may be visible around the eye.

**C. Minimum criteria:** The IOT appears shorter and/or bulging.

*AU43—eye closure and AU45—blink*. In the **human** FACS, the distinction between blinks and eye closure is temporally based, where AU45 is coded if the eye is closed for half a second or less, while AU43 codes varied intensities of the lower eyelid moving over the eyeball to close the eye, with AU43E (E denoting maximum intensity) coding complete closure of the eye (upper eyelid touching lower eyelid and covering the eyeball completely). Unlike all other AUs where a muscle contracts to produce movement, the closure of the eye or blink by AU43/45, respectively, is produced by the relaxation of the levator palpebrae muscle, a thin sheet of muscle enveloping the eyeball cranially. In **marmosets** the levator palpebrae has not been described (although it has been described in a closely related species, the Goeldi marmoset [69]), but it has a similar concentric orbicularis occuli muscle with the palpebral and the orbital portion.

In **humans**, blink rate ranges from 15 blinks/min [70] to 20.8 blinks/min [71] depending on the task, typically ranging between 200 and 500ms (average duration: 403.6 ms) [63]. In free moving **marmosets**, mean eye blinking is about 5.4 blinks/min and lasts on average 178.2ms [63]. Hence, since marmosets produce fewer blinks, but blink approximately two times faster than humans, the temporal definition for marmosets was adjusted accordingly.

**A. Proposed muscular basis:** Orbicularis occuli (pars palpebralis) and possibly levator palpebrae relaxation.

**Fig 9:**
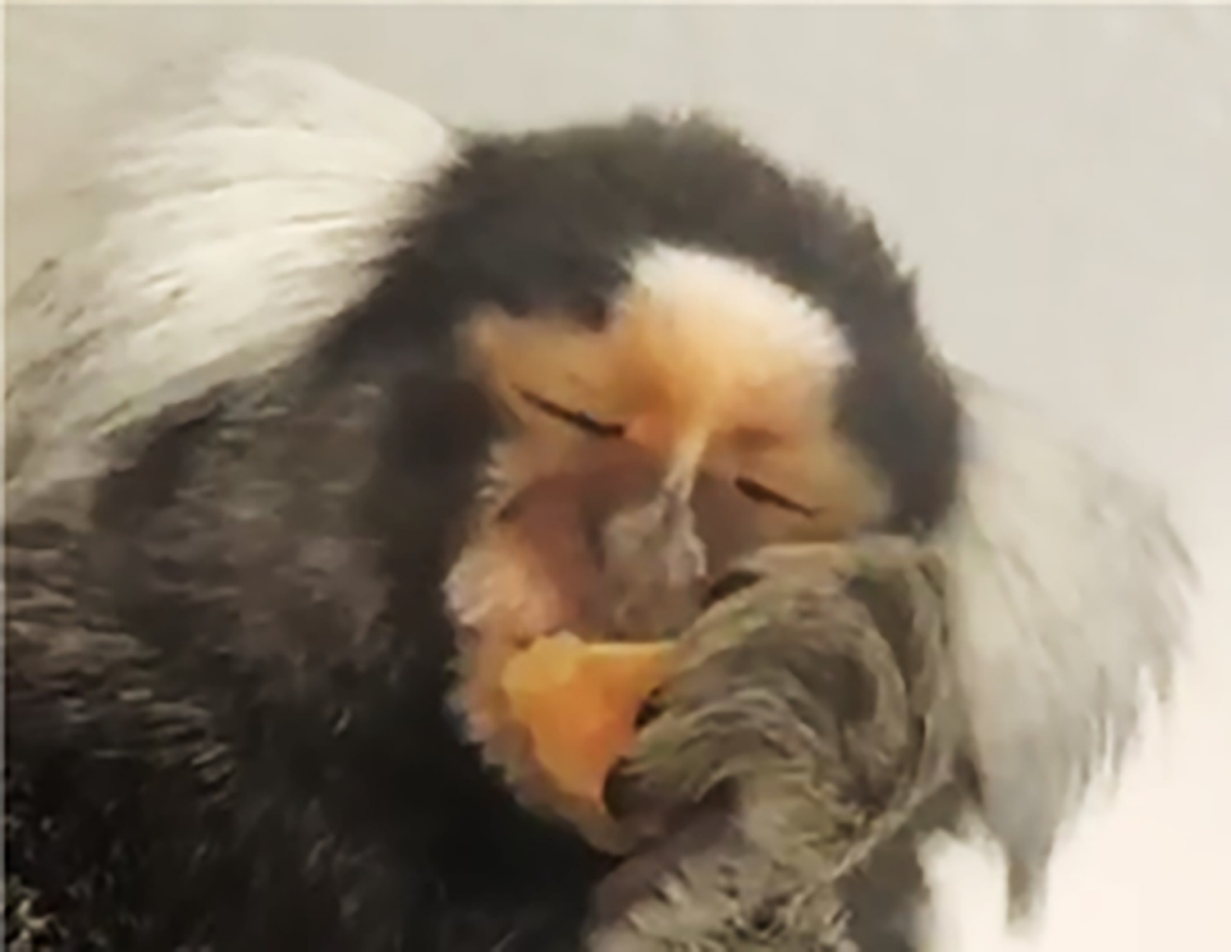
AU43—eye closure/AU45—blink at the apex with appearance change 3.

**B. Appearance changes**:

1. The upper eyelid moves towards the lower eyelid, reducing the eye opening until it closes the eye completely (i.e. the eyelids cover the eyeball completely).
2. The upper eyelid (cream coloured) becomes more visible.
3. When the eyelids touch each other closing the eye without additional tension from pressing eyelids against each other, a thin black line can be seen (**Fig 9**).
4. Subtle movement or tension on the skin and hairs might be seen globally around the eyes, including browridge, IOT and both eye corners.
5. The lower eyelid might display subtle movement towards the upper eyelid.
6. Unilateral movements or AU43/AU45 with different onsets/offsets and apex can occur.
7. All appearance changes above can be observed in both AU43 and AU45. However, in **AU43** the eye remains closed for 250ms or more (**S9** Video), while in **AU45** the eye opens within 250ms (**S10a** and **S10b** Video). Therefore, AU43 and AU45 are mutually exclusive, as they cannot be coded simultaneously.

**C. Minimum criteria:** the eyelids move towards each other (or only upper eyelid) and cover the eyeball completely.

**D. Subtle differences between AUs:** In the human FACS, appearance changes 3 and 4 are part of AU6 and AU7—Lid Tightener (lower eyelid is raised or bulged), respectively. AU7 is not observed in marmosets in isolation, but AU6, although rarely observed, is present without AU43/45. Nonetheless, since the appearance changes of AU6 are frequently accompanied by AU43/45, consider coding AU6 as well, whenever the IOT is shortened or bulged.

*AU47—Half-Blink*. The AU47 –The Half-Blink has not been described by Ekman and colleagues [3] in the **human** FACS, but has been described for the domestic cat in the CatFACS [16]. This movement is frequently observed as part of the behavioural repertoire of the domestic cat and is described as sequential movements of the eyelids towards and away from each other, without ever closing the eye completely. This movement has been observed in other species (e.g. horses [15]; dogs: [72]) as a single movement instead of a sequence of movements. Although the function of this movement in marmosets is still not clear, in humans it has recently been linked to differences in spontaneous versus voluntary movements [73] and in cats seems to function in communicating positive emotion towards humans [74]. In **marmosets** the single AU47—Half-Blink, is described below (S11a, S11b, S12a, S12b, S13a and S13b Videos).

**A. Proposed muscular basis:** Orbicularis occuli (pars palpebralis) and possibly levator palpebrae relaxation.

**B. Appearance changes**:

1. The upper eyelid moves towards the lower eyelid, reducing the eye opening and returning to neutral without ever touching the lower eyelid.
2. In some movements, both upper and lower eyelids are seen moving towards and away from each other.

**C. Minimum criteria:** the eyelids move towards each other (or only upper eyelid), but do not cover the eyeball completely (eyelids might touch near the eye corners, but not medially).

**Fig 10:**
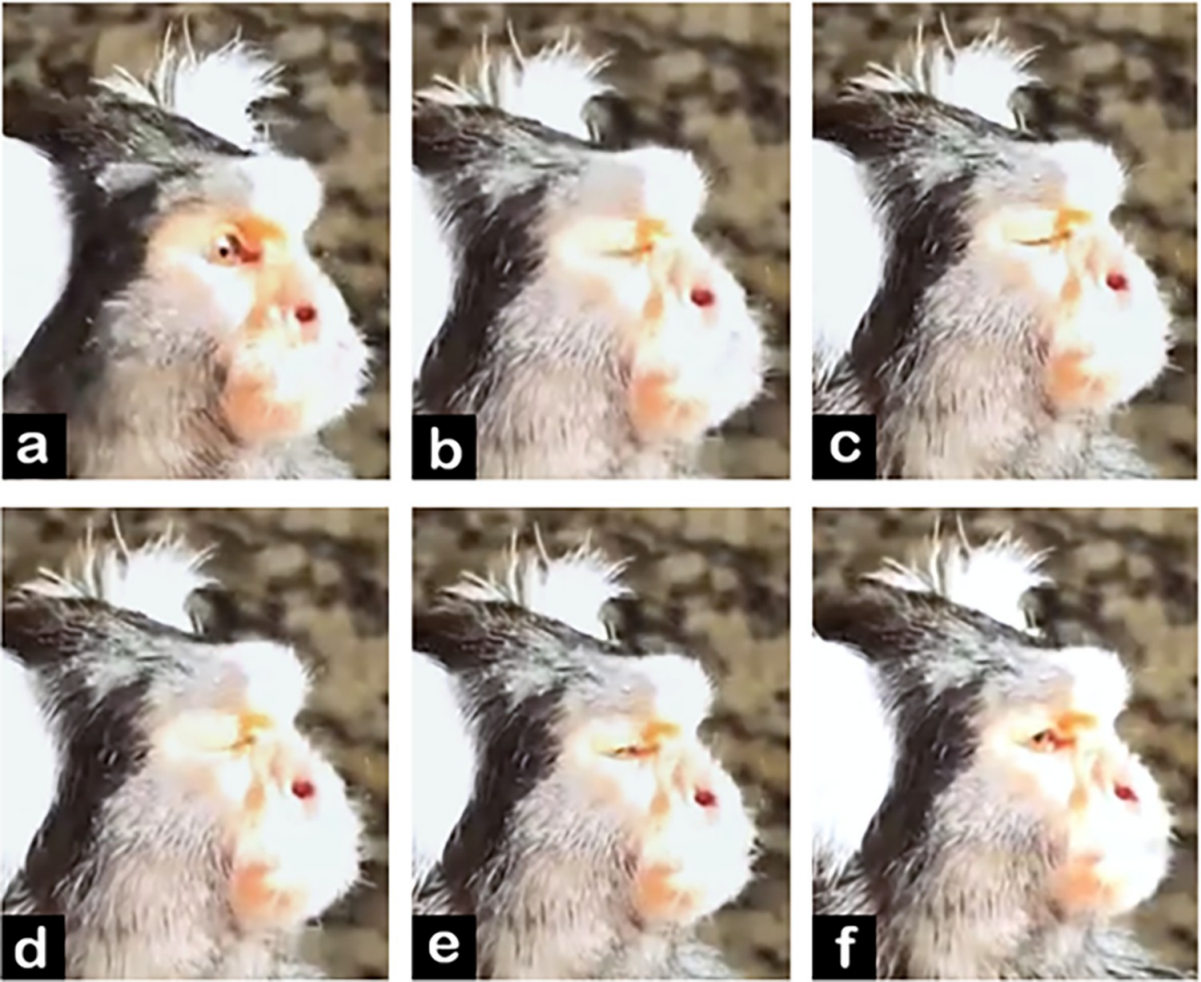
Examples of eyelid position at the apex of AU43/45 or AU47. a) eyelids do not cover eyeball, and thus AU43/45 and AU47 are not present. b), c) and d): eyelids cover eyeball completely at the apex of the eyelids movement, and thus AU43/45 is coded. e) and f): eyelids cover eyeball only partially at the apex, and thus AU47 is coded. Other AUs are present (e.g. AU41).

**D. Subtle differences between AUs:** AU47 is coded when the eyelid(s) move towards each other without fully covering the eyeball, while AU43/45 is coded whenever the eyeball is completely covered by the eyelids (**Fig 9**). With AU47, no movement around the eye seems to be displayed, so if any further movement is detected in the browridge or IOT consider coding AU41 or AU6, respectively. Additionally, the eyelids might present slight movement when the individual changes eye direction, but this should not be coded as AU47. See Fig 10 for examples of differences between AU43/45/47.

**Fig 11:**
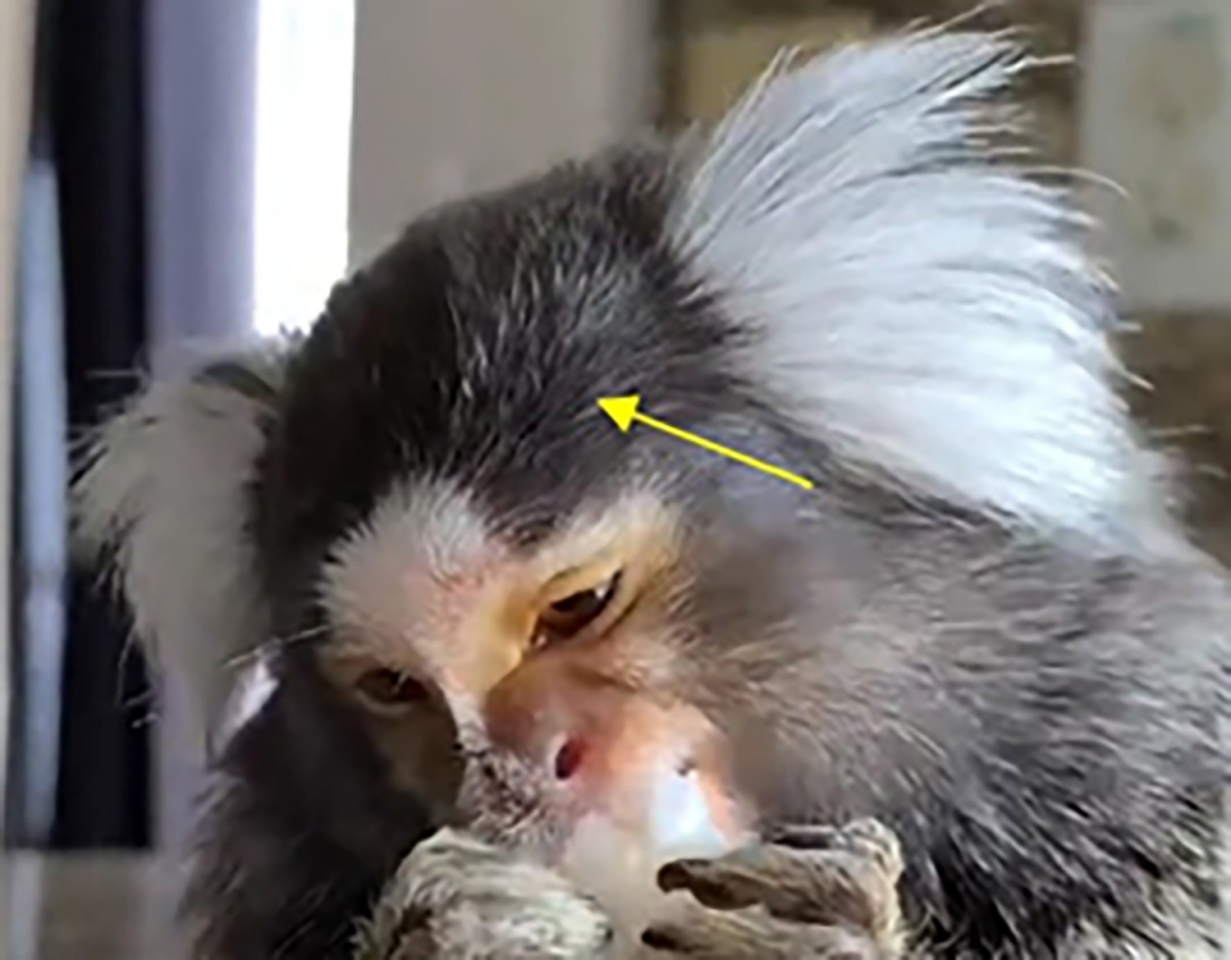
AU109+110—Nose Wrinkler + Upper Lip Raiser. The arrow indicates the direction of movement that is seen when the upper lip is pulled towards the inner eye corner.

**Lower face action units.** *AU109+110—Nose Wrinkler + Upper Lip Raiser*. In **humans**, AU9—Nose Wrinkler, and AU10—Upper Lip Raiser, are coded independently. In AU9, the levator labii superioris alaeque nasi muscle wrinkles the nose and in AU10, the levator labii superioris muscle raises the upper lip. In **marmosets**, due to morphological (e.g. prognathism) and anatomical differences (e.g. less muscular differentiation and different relative position of the muscles), AU9 appearance changes always overlap with some of the AU10 appearance changes, and therefore AU9 is coded together with AU10. Furthermore, since in marmosets the underlying musculature producing these movements is not homologous to the human musculature (**Table 1**), and to follow coding nomenclature from previous AnimalFACS (e.g. CatFACS [16]), the AU codes have been slightly modified to reflect the underlying musculature. The levator labii superioris pulls the medial portion of the lip dorsally and wrinkles the nose, while the superior auriculolabialis pulls the lateral portion of the lips dorsally. Thus, in marmosets, AU109+110 is coded as a combined movement (**Fig 11, S14A, S14B, S15**, **S16A** and **S16B** Videos).

**A. Proposed muscular basis:** Levator labii superioris, superior auriculolabialis.

**B. Appearance changes**:

1. The upper lip is pulled dorsally with a clear oblique movement towards the inner eye corner (**Fig 11**).
2. The nasal wing is also pulled dorsally and it may widen.
3. In stronger movements, wrinkles form on the inner eye corner, running laterally on the nose (**S14A** and **S14B** Video).
4. Both the lower eyelid furrow and the infraorbital furrow deepen.
5. The skin bulges on the IOT.
6. The teeth may become exposed.

**C. Minimum criteria**: the upper lip is pulled towards the inner eye corner or the nasal wings are pulled dorsally.

**D. Subtle differences between AUs:** In very strong movements of AU109+110, the eye aperture may be narrowed, the glabella pulled ventrally, and wrinkles formed on the inner eye corner running laterally to the nose (**S14A** and **S14B** Video). In these instances, most of the appearance changes overlap with AU41; if the minimum criteria for AU41 is present, then it should also be coded. In very strong AU109+110, AU6 might be present as well, due to constriction of the eye. Therefore, AU6 should also be coded.

*AU110—Upper Lip Raiser*. Although in **marmosets** AU109 is always accompanied by AU110, AU110 is also observed on its own, raising the lip in a different direction than AU109+110 and creating different appearance changes (**S17A, S17B, S18A, S18B, S19A and S19A Videos**). As only the more lateral portion of the lip was observed being raised, AU110 in marmosets is likely due to the action of the superior auriculolabialis muscle. AU110 can also be produced by the zygomaticus minor on the lateral portions of the lip (near the lip corners), as seen in **S19a** and **S19b** Video, or in the medial portion of the lip by the action of the LLS muscle (producing AU109+110).

In **humans**, AU10 is an up/down movement, due to the positioning of the muscle and the direction of contraction. In **marmosets**, the anatomical position of the muscles which produce AU110 is oblique, where both the superior auriculolabialis and the zygomaticus minor likely act to raise the upper lip towards the outer corner of the eyes or towards the ears. The zygomaticus minor seems to vary broadly in terms of its presence in primates, but it has been identified in both humans and in marmosets.

**A. Proposed muscular basis:** Superior auriculolabialis, zygomaticus minor.

**Fig 12:**
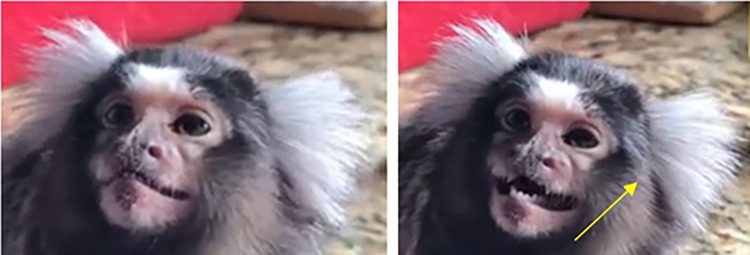
AU110—Upper Lip Raiser. The arrow indicates the direction of movement when the upper lip is pulled towards the ears.

**B. Appearance changes**:

1. The more lateral portions of the upper lip are pulled dorsally with a clear oblique movement towards the ears (**Fig 12**).
2. The nasal wing is also pulled dorsally and it may widen the nose.
3. The nasiolabial furrow deepens and the IOT might bulge slightly.
4. The teeth are usually exposed (at the least the canines tip becomes visible).

**C. Minimum criteria**: The upper lip lateral portions are pulled towards the ears.

**D. Subtle differences between AUs:** The main difference between AU109+110 vs. AU110 is the direction of the dorsal movement, which in the former is towards the inner eye corner, by the LLS muscle, and in the latter is towards the ears, by the action of the SAL/zygomaticus minor muscles.

*AU12—Lip Corner Puller*. In a neutral face of marmosets, the lip corners extend backwards until the edge of the lighter area of the face, ending straight in some individuals and in others presenting a slight downwards curvature. This "curved" appearance of the lip corners is a false indicator and can change depending on the point of view of the observer. Therefore, additional caution regarding the movement of the lip corners and careful comparison with the neutral face is needed. The lip corner puller is produced by the zygomaticus major muscle, both in **humans** and **marmosets**, pulling the lip corners back (**S20A, S20B, S21A, S21B, S22A and S22B** Videos).

**A. Proposed muscular basis:** Zygomaticus major.

**Fig 13:**
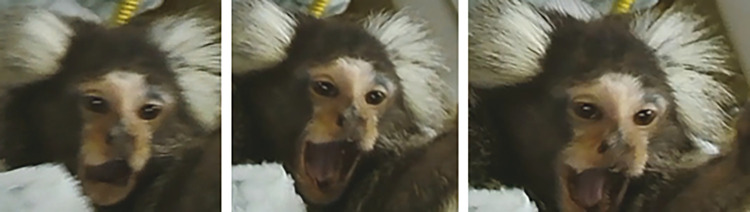
AU12—Lip Corner Puller. Left: AU25 and AU27 are present; Centre: AU12 is added to AU25+AU27; Right: AU110 is added to AU25+AU27+AU12.

**B. Appearance changes**:

1. The lip corners are pulled dorsally.
2. In less intense movements, AU12 might be visible only in the lip corner area (**S20A** and **S20B** Video). In more intense movements, the skin of the upper and lower lip and mental region is stretched and flattened, with movement that can be detected along the lips until the medial part.
3. In more intense movements, the philtrum flattens and widens slightly (**Fig 13**, **S21A** and **S21B** Video).
4. Some wrinkling/bunching of the skin might form around the lip corners.
5. Movement of the hair dorsal to the lip corners may also accompany the movement.
6. The lips may part in more intense movements and expose the upper teeth.
7. AU12 is frequently observed simultaneously with AU26/AU27 (**Fig 13**). When AU26/AU27 is produced with AU12, the lip corner may also appear to curve slightly upwards.

**C. Minimum criteria**: The lip corners are pulled dorsally.

**D. Subtle differences between AUs:** When AU26 or AU27 are acting, particularly with AU27, the lips might slide caudally due to the mouth aperture which may give the impression of AU12 due to the lip corner angle (**Fig 13**). However, only code AU12 if a pulling movement is observed independently on the lip corner area. AU12 will deepen the lip corners when acting (**Fig 13**). When AU110 is produced on the more lateral part of the upper lip, it can be confused with AU12. However, the direction of the movement is different. With AU12 only, there is no dorsal movement of the upper lip.

**Fig 14:**
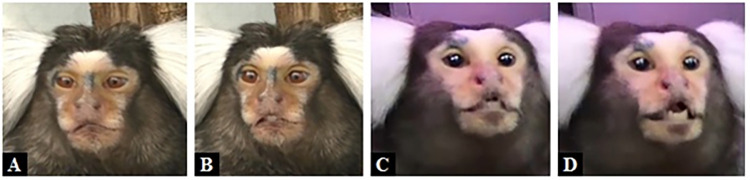
AU16—Lower Lip Depressor. **A**: Neutral; **B**: AU16 pulls the lower lip downwards medially; **C**: AU16 pulls the lower lip downwards medially and laterally; **D**: AU16 pulls the lower lip further down (AU12 also present).

**Fig 15:**
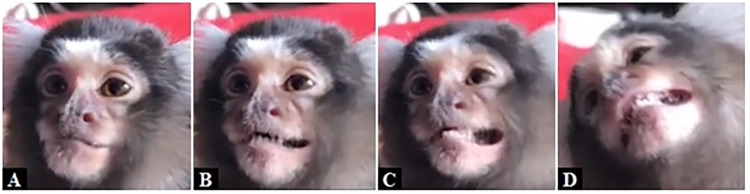
AU16—Lower Lip Depressor. **A**: Neutral; **B**: AU16 pulls the lower lip downwards medially and laterally (AU12 also present); **C**: AU16 pulls the lower lip further down laterally (AU110+AU12 also present); **D**: AU16 pulls the whole lower lip further down (AU110+AU12 also present).

*AU16—Lower Lip Depressor*. The Lower Lip Depressor—AU16—in **humans** and **marmosets** is produced by the same muscle, and due to the lip morphology it presents similar appearance changes (**Figs 14** and **15; S23A, S23B, S24A, S24B**, **S25A** and **S25B** Videos). This movement only occurs after parting of the lips (AU25).

**A. Proposed muscular basis:** Depressor labii inferioris, platysma might also be recruited to pull down more lateral portions of the lower lip.

**B. Appearance changes**:

1. The lower lip is pulled downwards, sliding over the mental region and parting the lips (hence AU16+AU25 is usually coded).
2. The convex curvature of the lower lip softens and becomes flatter.
3. It may expose the lower teeth.
4. The mental region decreases in apparent size.
5. The inner part of the lower lip becomes more visible.
6. The skin of the mental region may flatten/stretch.
7. In low intensity movements, the cues are more apparent in the medial region, while in more intense movements the whole lower lip slides downwards, giving a squared shape appearance to the lower jaw and mental region (**Figs 14** and **15**).

**C. Minimum criteria:** The lower lip is pulled downwards either in the medial portion, the lateral portions or both.

**D. Subtle differences between AUs:** In previous literature [45] broad facial expressions descriptions featured lip corners turned downwards. In humans, lip corners are pulled down by AU15—Lip Corner Depressor, activated by the depressor anguli oris muscle. In **marmosets**, this muscle is also present, but no correspondent AU15 is observed. However, due to the lower medial lip curving slightly upwards (i.e. shaped as) on a neutral face, the lip corners may appear to be pulled downwards, giving the false indicator of AU15 acting. Furthermore, it is possible that in marmosets, the depressor anguli oris pulls the lower lip downwards laterally instead of turning the lip corners downwards. However, further research is needed to understand the exact function of the depressor anguli oris muscle.

**Fig 16:**
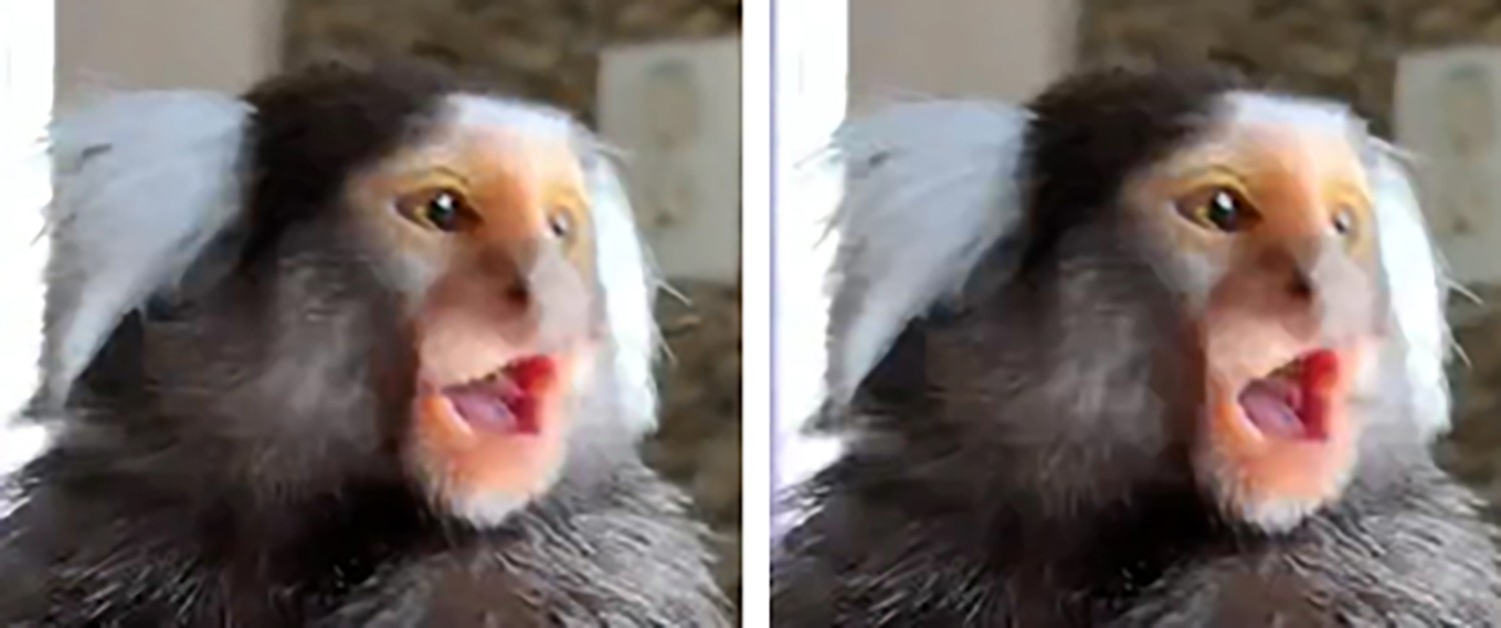
AU118—Lip Pucker. Left: AU25 and AU27 are present; Right: AU118 is added to AU25+AU27.

**Fig 17:**
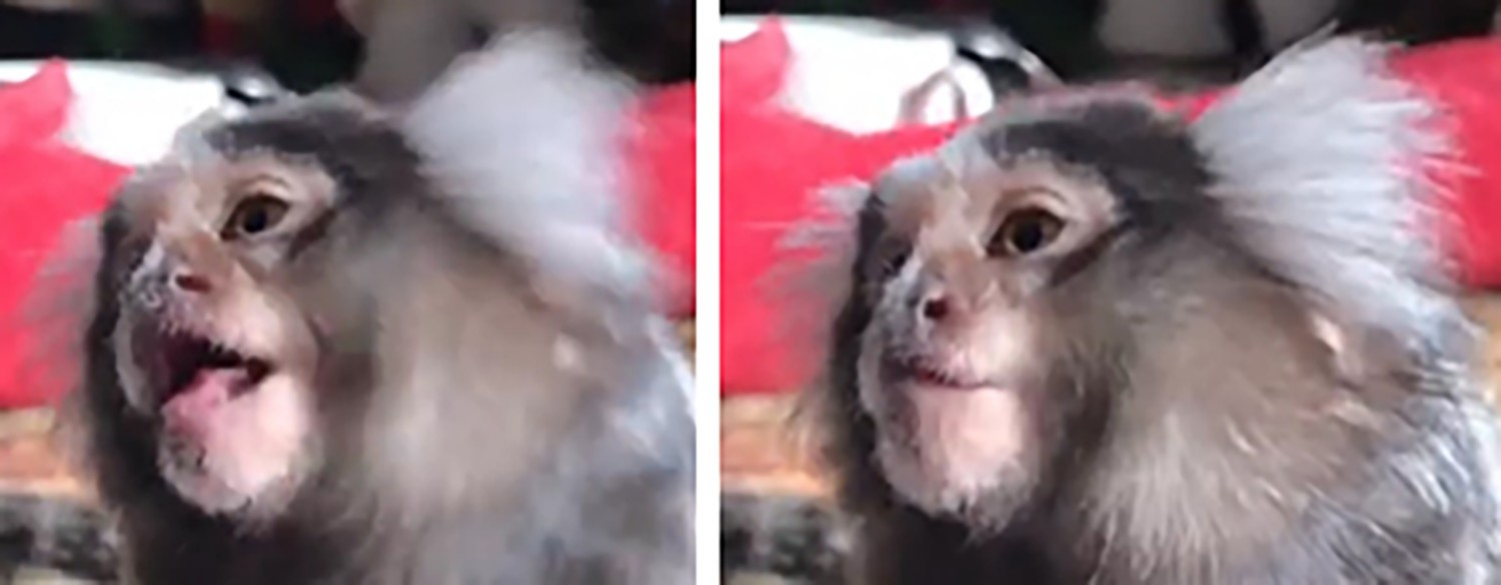
AU118—Lip Pucker. Left: AU25 and AU27 are present; Right: AU118 is added after mouth closure.

**Fig 18:**
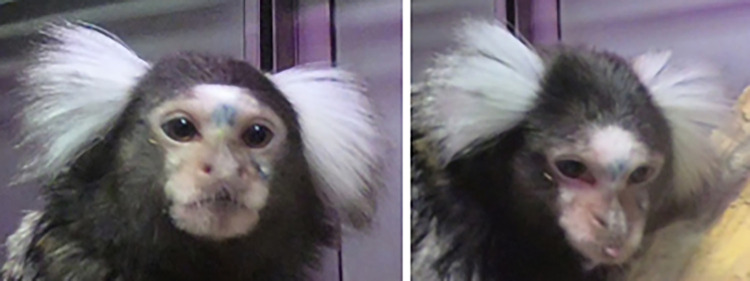
AU118—Lip Pucker. Left: Neutral; Right: AU118 is present during AD19—Tongue Show.

*AU118—Lip Pucker*. In humans, AU18—Lip Pucker is produced by the incisivii labii muscles that bring the lips together medially, moving the lip corners towards each other, and puckering them. These muscles were not identified in marmosets and they do not present the characteristic puckering action observed in humans with the wrinkling of the lips. However, in marmosets, a similar movement without wrinkling or puckering was observed, where the lip corners are drawn medially by the action of the orbicularis oris and the buccinator muscles (with the latter used to keep food between the molars during chewing). Hence, in marmosets AU118 is used to code the movement of the lip corners towards the medial region of the mouth (Figs 16–18; S26A, S26B, S27A, S27B, S28A, S28B, S29A and S29B Videos).

**A. Proposed muscular basis:** Orbicularis oris and buccinator.

**B. Appearance changes**:

1. The lip corners are pushed towards the mouth midline.
2. Some medial bulging of the lips may be observed.
3. The mouth area may appear to become narrower as it appears compressed medially.
4. If the mouth is open, fewer teeth may become visible. The sharp angle of the lip corners becomes rounder (**Fig 16**).
5. Skin and hair accompany the lip corner movement towards the medial area of the mouth.

**C. Minimum criteria:** The lip corners are pushed medially.

**D. Subtle differences between AUs:** AU118 is mutually exclusive to AU12 as they are movements in opposing directions. However, AU118 might be confused with AU12 returning to neutral and vice-versa. Therefore, identifying the neutral position of the lip corners in a particular individual is important to distinguish these two movements and the release of the opposing movement.

*AU25—Lips Part*. In **humans** and **marmosets**, AU25 codes the separation of the lips, i.e. it is coded whenever a space is observed anywhere between the upper and lower lip (**S30a** and **S30b** Video). This AU can be caused by many different muscles. Unlike other AUs, AU25 can be caused by other AUs and is usually coded with the AUs that caused the lips to part (if there are enough appearance changes to identify the other AUs). For example, AU110 alone might cause AU25, in which case AU25+AU110 are coded.

**A. Proposed muscular basis:** A range of muscles attaching to the lips might produce this movement, including orbicularis oris, levator labii superioris, depressor labii inferioris, etc. Jaw opening also can produce AU25.

**B. Appearance changes**:

1. The lips are separated at any point and a space can be observed between the upper and lower lip.
2. The inner lips or teeth may become visible.

**C. Minimum criteria:** The lips part and a space is observed between the lips.

*AU26—Jaw Drop*. In **humans** and **marmosets**, AU26—Jaw Drop describes a movement produced by the relaxation of jaw muscles (masseter and temporalis). These non-mimetic muscles open the jaw slightly by relaxing (**S31A, S31B**, **S32A** and **S32B** Videos). At rest, the jaw muscles are contracting to keep the jaw closed and in a neutral position.

**A. Proposed muscular basis:** Masseter and temporalis muscles relaxation.

**B. Appearance changes**:

1. The lower jaw is lowered and teeth separation can be clearly seen or inferred.
2. This jaw lowering is a relaxation movement, of small amplitude, where no sign of tension or skin stretching around the mouth is visible.
3. If the lips part (if AU25 is also present), teeth might become visible. However, AU26 can occur without lip parting.

**C. Minimum criteria:** The lower jaw moves downwards in a small and relaxed movement.

**D. Subtle differences between AUs:** AU25 and AU26 are dorsoventral movements of the mouth, but AU25 refers only to the parting of the lips, while AU26 refers to the parting of the jaw. They can be coded independently, as it is possible that the lips are parted without parting the jaws and, conversely, the jaw might be slightly opened without the lips parting. In the case that both lips and jaws are parted, AU25+AU26 is coded. Code AU26 also when the jaw is parted due to an object keeping the jaw separated (e.g. food held between upper and lower teeth, tongue placed between upper and lower teeth). In still images, a weak AU26 might be difficult to code if there is no clear separation of the teeth.

*AU27—Mouth Stretch*. In **humans** and **marmosets**, AU27—Mouth Stretch, describes a movement produced by the pulling of the lower jaw downwards (by non-mimetic muscles), stretching the mouth wide open. Although AU27 describes a larger degree of mouth opening in relation to AU26, AU27 is mutually exclusive to AU26, as different muscles are involved.

**Fig 19:**
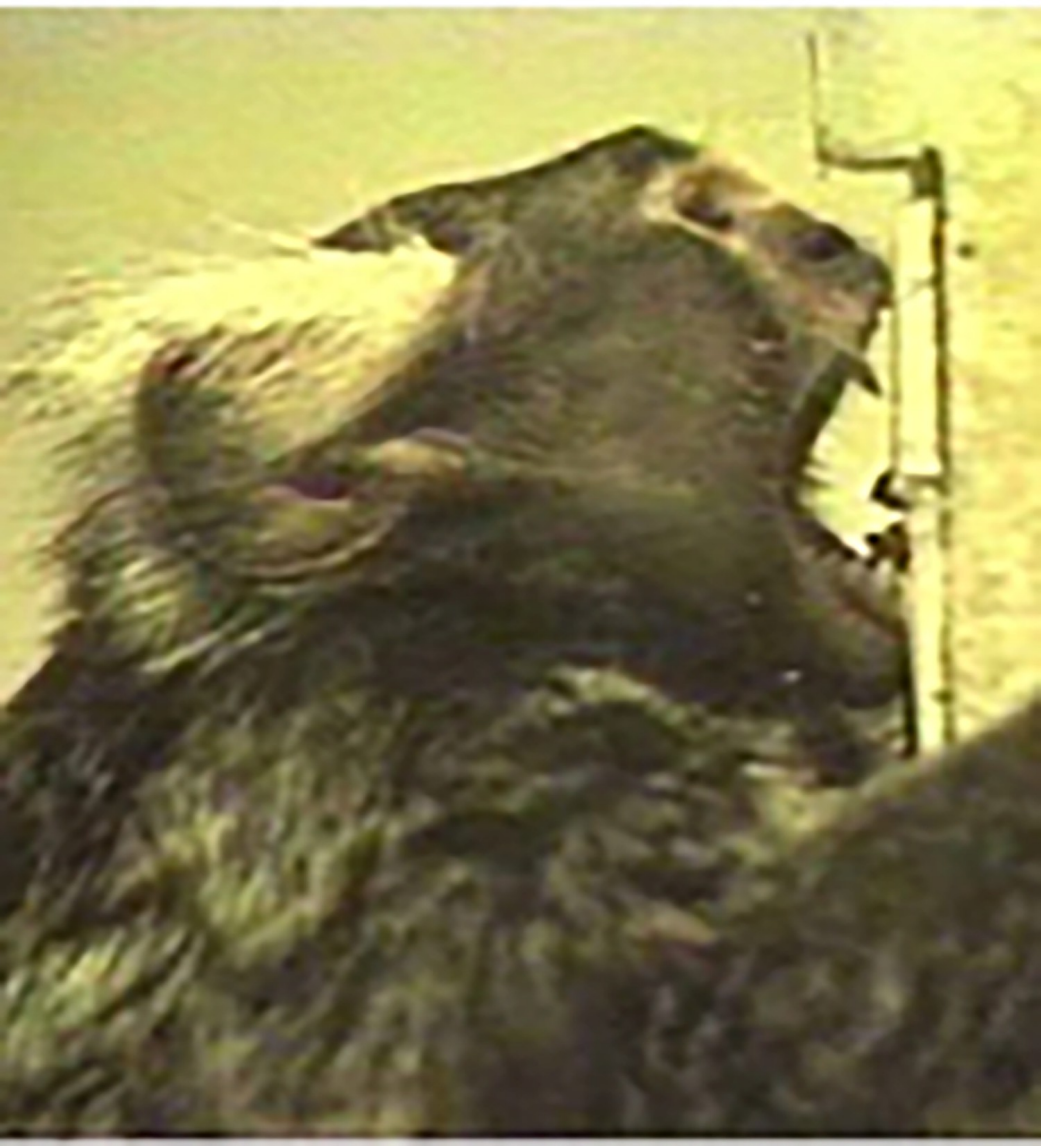
Tree gouging action with AU27—Mouth Stretch at its apex (adapted from [76]).

**Marmosets** feed from tree exudate (emitted gum), anchoring their upper jaw into the tree substrate while using their lower jaw to remove bark and extract exudate [75]. During this tree gouging, marmosets are able to open their jaws in a very wide gape up to 25mm [23, 76] (**Fig 19**), due to long fibres of the masseter muscle and other anatomical specifications that allows long stretching of the jaw [75]. However, during other jaw movements the mouth gape is comparatively much smaller, averaging at 10 mm [23] (**S33A, S33B, S34A, S34B**, **S35A** and **S35B** Videos). Nonetheless, marmosets are able to produce a large AU27 due to their jaw anatomy.

**A. Proposed muscular basis:** The non-mimetic muscles that open the jaw include the anterior belly of the digastric muscle, the mylohyoid muscle, and the inferior head of the lateral pterygoid muscle. These muscles are likely very well developed in marmosets producing their unusually large gape during gouging.

**B. Appearance changes**:

1. The mouth is opened wide by lowering the lower jaw and actively stretching it downwards.
2. The lips become stretched.
3. The lower teeth, tongue and oral cavity are exposed. The upper teeth may also be exposed.
4. The global shape of the face becomes dorsoventrally extended.
5. In large movements, the lips may retract as the opening of the mouth forces the lips to slide caudally.
6. AU27 must be coded together with AU25 as there is always lip separation due to the degree of mouth opening.
7. Often combined or immediately followed by other AUs.

**C. Minimum criteria:** The mouth is stretched open, further than jaw relaxation (AU26), with clear signs of skin stretching.

**D. Subtle differences between AUs:** Although AU26 and AU27 describe visually different degrees of mouth opening, and are produced by different muscles and different muscle actions (relaxation and contraction, respectively), these AUs are mutually exclusive. A wide AU26 and a weak AU27 can be confused, especially in still images. Careful examination of the movement itself, noticing stretching and tension around the mouth and lips, helps in deciding if the jaw is being relaxed or actively pulled down. In a strong AU27, AU12 is also often present, stretching the mouth laterocaudally and further increasing tension and stretching around the mouth, nose and infraorbital region. AU110 and AU16 are also frequently observed together with AU27. On the other hand, when AU25/26/27 act, the upper and/or lower teeth may become visible and produce a false indicator as if AU110 or AU16 is acting. However, code AU110 or AU16 only if there is clear movement of each lip upwards or downwards, respectively, and/or different onsets/offsets are observed. AU26 and AU27 might also be alternated, where a temporal distinction might be useful to decide when to code AU26 as an independent movement, or not to code it as it is produced as a precursor to AU27. For example, if AU26 is held for a couple of seconds, and then AU27 follows, then both AU26 and AU27 should be coded. Alternatively, for each instance of mouth opening, only the highest degree of opening might be coded, which means that if an AU27 follows AU26 without mouth closure, only AU27 is coded regardless of AU26 duration.

*AU38—Nostril Dilator*. **A. Proposed muscular basis:** Nasalis.

**B. Appearance changes**:

1. The nostril wing moves outwards.
2. The nostril increases in size and might change shape.
3. The skin next to the nose might move.
4. In some movements, the nose may appear to flatten.

**C. Minimum criteria:** The nostril wing moves outwards and/or the nostril is widened.

**D. Subtle differences between AUs:** Caution regarding appearance change 3 is needed, as movement of the skin around the nose might be due to other AUs pulling the skin globally in the mouth region (e.g. AU110, AU16). AU38 is coded only if one the minimum criteria are present: movement of the nostril wing outwards or widening of the nostril area. Additionally, AU38 may be difficult to detect depending on the angle and distance of the individual from the camera. Changes in head position might appear to change the nostril shape. Therefore, if there are head movements that can change the observed nostril shape, AU38 should be coded only when movement is detected in the nostril wing.

### Action descriptors

**Action Descriptors (ADs)** identify and describe more broad muscular movements or movements from non-mimetic muscles. The muscles responsible for ADs are not innervated by the facial nerve (cranial nerve VII [77]). These movements are often observed in combination with AUs, altering their visual appearance. Hence, section “A. Proposed muscular basis” is omitted below for all ADs, as either no movements are related to facial musculature or the musculature has not been clearly identified. In addition, section “D. Subtle differences between AUs” is included below where necessary, to help distinguishing between similar ADs or AUs. Other miscellaneous movements classified as ADs including head and eye direction, gross behaviours, and visibility status are briefly described in **S1 Text**.

**AD19—Tongue Show. B. Appearance changes**:

1. The tongue is extended outwards and reaches at least the inner lower lip (**S38A** and **S38B** Video).
2. The jaw must be lowered and the lips are separated. Therefore, this AD is always coded together with AU25+AU26 or AU25+AU27.

**C. Minimum criteria:** The tip of the tongue touches the lower lip.

The tongue of marmosets can extend far outside the mouth and can fold upwards and downwards, with the tip able to fold in a different direction as well. Hence, AD190 and AD191 are used to code different tongue movements outside the mouth.

**AD190—Tongue Downwards. B. Appearance changes**:

1. The tongue is extended beyond the lips and folded ventrally (**S39A** and **S39B** Video).
2. The ventral part of the tongue can be seen touching/licking the mental region.
3. The jaw must be lowered and the lips are separated. Therefore, this AD is always coded together with AU25+AU26 or AU25+AU27.

**C. Minimum criteria to code AD190:** The tongue is projected beyond the lips and folds downwards.

**D. Subtle differences between AUs/ADs:** This AD is distinguished from AD19 due to a clear ventral movement of the tongue (forming a curve with the tip of tongue pointing downwards), while in AD19 the tongue is only seen in a craniocaudal axis motion (no curvature is observed, tip of the tongue points forward). AD19 and AD190 are mutually exclusive (i.e. each time the tongue is projected beyond the lower lip, only one of these movements can be coded).

**AD191—Tongue Curl. B. Appearance changes**:

1. The tongue is extended beyond the lips and is curled upwards (**S40A** and **S40B** Video).
2. The jaw must be lowered and the lips are separated. Therefore, this AD is always coded together with AU25+AU26 or AU25+AU27.

**C. Minimum criteria to code AD191:** The tongue is projected beyond the lips and folds or curls upwards.

**D. Subtle differences between AUs/ADs:** This AD is distinguished from AD190 by the direction of movement of the tongue: in AD190 the tongue is projected downwards, while in AD191 the tongue is projected out and upwards. AD19, AD190 and AD191 are mutually exclusive (i.e. each time the tongue is projected beyond the lower lip, only one of these movements can be coded).

**AD29—Jaw Thrust. B. Appearance changes**:

1. The jaw is pushed forwards.
2. The mental region becomes more prominent and may bulge slightly.
3. If the mouth is open, the lower teeth are seen moving cranially (**S41A** and **S41B** Video).
4. The lower face appears rounder from a profile view (**S42A** and **S42B** Video).

**C. Minimum criteria to code AD29:** The jaw is projected forward.

**D. Subtle differences between AUs/ADs:** There may be individual variation in the relative position of upper and lower jaws in the marmoset. The mandible may be naturally positioned further forwards or backwards in relation to the maxilla and so AD29 should only be coded when movement is observed. Therefore, this movement cannot be coded from still images.

**AD30—Jaw Sideways. B. Appearance changes**:

1. The jaw is moved laterally to one side in relation to the midline (**S43A, S43B**, **S44A** and **S44B** Videos).
2. If the mouth is open, the lower teeth are seen moving to one side.

**C. Minimum criteria to code AD30:** The jaw is moved to one side of the face.

AD181—Lip Smacking. B. Appearance changes:

1. The lips are rapidly and repeatedly moved towards one another (**S45A** and **S45B** Video).
2. The mental region might also move repeatedly dorsoventrally.
3. It may be accompanied by vocalisations or be silent.
4. It is usually accompanied by AU118 or AU25.

**C. Minimum criteria to code AD181:** A rhythmic movement of the lips towards each other must be observed. Therefore, this movement cannot be coded from still images.

### Ear action descriptors, tufts and scalp movements

In **humans**, three external ear muscles (auricularis superior, posterior and anterior) are present, but movements are not observed in all individuals. When humans are able to move their ears voluntarily, these movements are usually of small amplitude and seem to be non-functional [78], with most ear muscle activity reflexive and non-observable [79]. In **marmosets**, well defined ear muscles which are homologous to the human ones have been identified. Ear movements may be related to ear orientation towards a wide range of conspecific vocalisations [80], but may also be important for visual communication [41], as the ears are surrounded by conspicuous white tufts of hair that attach around the pinna base. However, there is no evidence that the tuft movement is produced by dedicated muscles (i.e. unlike vibrissae that have intrinsic muscles for independent movement of each hair). It is possible that tuft movement is derived from the skin moving when other muscles contract (e.g. frontalis, occipitalis, platysma) or from ear movement itself. However, tuft movement is also observed independently of ear movement. An additional difficulty in identifying ear movement is related to the relative position of the ears and tufts, where the tufts obscure the shape of the ears in frontal views of the marmoset head. The underlying musculature producing tuft and ear movement is not yet clear (thus indicated as "unknown" in **Table 2**), and the neutral position of the ears and tufts is difficult to determine. Therefore, ear and tuft movements are described as ADs and EADs, respectively.

The EADs are movements from a neutral position (established a priori for each individual) due to the action of the ear musculature. The two ears display independent movements, and so unilateral EADs can be coded using left (L) and right (R), respectively for each ear (e.g. EAD1L, EAD1R). These codes are also used for homologous muscles in rhesus macaques (EAD1 and EAD3) in MaqFACS and for non-homologous muscles in dogs and cats (EAD105) in DogFACS and CatFACS. Other ear movements found in other species, such as EAD2 (Ears Elevator) found in rhesus macaques, and EAD104 (Ears Rotator) and EAD106 (Ears Backwards) found in dogs and cats were not observed in marmosets.

The white tuft movements are described as ADs as the musculature producing these movements is unclear. Scalp Retraction (AD101), previously described in crested [81] and Japanese [11] macaques was also observed in marmosets and encompasses both ear and tuft retraction.

**EAD1—Ears Forward. A. Proposed muscular basis:** Anterior auricularis pulls the ear cranially.

**B. Appearance changes**:

1. The ears move cranially (small amplitude movement, **S46a, S46b, S47a** and **S47b** Videos).
2. The tufts and hair around the pinna may accompany this movement.

**C. Minimum criteria:** The ears move forward.

**D. Subtle differences between AUs:** EAD1 is a very small amplitude movement. Therefore, it is the most difficult ear movement to detect in marmosets and is only observed when the individual directs its attention towards an event. It also might be difficult, or impossible, to detect from a frontal view. Therefore, if the ears are not visible, ear visibility codes may be useful to account for ears which are out of view (**S1 Text**).

**EAD3—Ears Flattener. A. Proposed muscular basis:** Posterior auricularis retracts the ear and flattens it against the head.

**B. Appearance changes**:

1. The ears move caudally, and in stronger movements the ears flatten against the head (**S48A, S48B, S49A** and **S49B** Videos).
2. The tufts and hair around the pinna might accompany this movement.

**C. Minimum criteria:** The ears move backward.

**D. Subtle differences between AUs:** A weak EAD3 may be confused with the release of EAD1 (Ears Forward) when the ear is returning to a neutral position. However, since EAD1 movements have very small amplitudes, it is usually possible to note at which point the ear goes beyond a neutral position to produce EAD3. Careful examination of the neutral position and the previous and subsequent movements will help to decide if an EAD3 should be coded.

**EAD105—Ears Downwards. A. Proposed muscular basis:** Depressor helicis muscle pulls the ear ventrally.

**B. Appearance changes**:

1. The ears are pulled ventrally (**S50A, S50B, S51A, S51B, S52A, S52B**, **S53A** and **S53B** Videos).
2. The tufts and hair around the pinna might accompany this movement.

**C. Minimum criteria:** The ears move downwards.

**D. Subtle differences between AUs:** EAD105 is rarely observed on its own and is often combined with EAD3 (**S52A, S52B, S53A** and **S53B** Videos). From a frontal view, it may not be possible to code this movement, either because the ear is completely out of view or because it may be difficult to distinguish from the Tufts Downwards (AD301) movement.

**AD300—Tufts Upwards. B. Appearance changes**:

1. The tufts move upwards (**S54A, S54B**, **S55A** and **S55B** Videos).
2. The hair within the tufts appears more spread out and larger in overall size, with a more oblique angle in relation to the head (in frontal view).
3. The distance between the tuft base in the frontal region might decrease.
4. The distance between the ventral portion of the tufts and the head might increase.
5. It can be asymmetrical, where movement is more evident in one tuft than the other.
6. It can be unilateral. Therefore, left (L) or right (R) AD300 can be coded (i.e. AD300L or AD300R).

**C. Minimum criteria:** The tufts move upwards.

**AD301—Tufts Downwards. B. Appearance changes**:

1. The tufts move downwards (**S56A, S56B, S57A, S57B, S58A, S58B, S59A, S59B**, **S60A** and **S60B** Videos).
2. The hair within the tufts appears less spread out and smaller in overall size, tending to be more parallel to the frontal region or lower than the frontal region (in frontal view).
3. The distance between the tuft base in the frontal region might increase.
4. The distance between the ventral portion of the tufts and the head might decrease as the tufts flatten against the head.
5. It can be asymmetrical, where movement is more evident in one tuft than the other.
6. It can be unilateral, with movement visible only in one of the tufts. Therefore, left (L) or right (R) AD301 can be coded (i.e. AD301L or AD301R, **S58a** and **S58b** Video).

**C. Minimum criteria:** The tufts move downwards.

**D. Subtle differences between AUs:** AD301 might be difficult to distinguish from a release of AD300, and vice-versa. AD300 tends to be a subtler movement than AD301. Therefore, the former is more difficult to code than AD301. Additionally, the tufts in marmosets present some slight individual variation in size, shape and coloration. Hence, for both of these movements, defining the neutral position of the tufts for each individual is crucial in order to maintain consistency in these codes and distinguish between them.

**AD101—Scalp Retraction. A. Proposed muscular basis:** In scalp retraction movements the occipitalis and posterior auricularis muscle actions work together as a unit to pull and flatten the ears, tufts and scalp towards the back of the neck. This muscle complex has previously been described in marmosets [52].

**B. Appearance changes**:

1. The skin of the frontal and occipital region is pulled caudally and then ventrally, where the tufts and ears are pulled towards the dorsal region of the neck (**S61A, S61B, S62A, S62B, S63A, S63B, S64A, S64B**, **S65A, S65B,** and **S66** Videos).
2. The distance between the tufts and eyes/glabella increases, the skin stretches on the lateral portion of the frontal region, and the hair direction accompanies the direction of the movement.
3. The head and face take a rounder appearance.
4. In stronger movements, the ears and tufts are flattened against the head and moved ventrocaudally.
5. In stronger movements, the skin around the whole face may be seen moving caudally, stretching and giving a rounder and larger appearance to the face.

**C. Minimum criteria:** The skin on the frontal region is pulled caudally, with simultaneous ear and tuft movement.

**D. Subtle differences between AUs:** AD101 is a movement produced after or simultaneously with both EAD3 and AD301. Therefore, whenever AD101 is coded it is not necessary to code EAD3 and AD301 as by definition they must be already acting beforehand. When coding the duration of AUs, if EAD3/AD301 start earlier than AD101, code the end of the former when the AD101 starts.

## Discussion and conclusions

In total, 15 AUs, 15 ADs and 3 EADs have been identified in common marmosets, which indicates a lower facial mobility than in humans (32 AUs), but similar mobility to other primates such as chimpanzees (15 AUs), orangutans (17 AUs), rhesus macaques (15 AUs), and gibbons (20 AUs). Importantly, the facial mobility in marmosets appears to be higher than previously thought, taking into account their socio-ecological characteristics [50, 51] as well as their primitive musculature [52]. The facial movements in marmosets also appeared to be faster than in other species, but more research is needed (i.e. a cross-species comparative study of AUs durations).

The common marmoset is a highly social species with a wide range of social behaviours (e.g. [42]). They make use of complex visual displays [43], including facial expressions [41]. To date, we lacked a scientific tool to measure facial movements in marmosets. However, the current methodological work, which aimed at identifying and classifying the full potential for movement in the common marmoset face, presents a new tool to allow its anatomical, objective, and standardised identification and measurement. For example, in humans "smiley faces" or "happy faces" can actually be teased apart into a wide range of different "smile" types that may not be linked to positive or even felt emotion [53], while recently in crested macaques, FACS was able to tease apart four variants of "silent-bared teeth" displays that are usually classified as only one display [81]. These studies demonstrate the highly complex and dynamic nature of facial expressions and highlight the need for FACS tools for the functional understanding of facial expressions. Likewise, for common marmosets, CalliFACS has the potential to identify and measure variation in their known facial expressions (e.g. "frown") to better frame possible functions, while avoiding anthropomorphic labels that may presume underlying emotions.

The CalliFACS also opens up the possibility of cross-species comparisons beyond the apes and Old World monkeys (e.g. orangutans [13], chimpanzees [8], macaques [9]), since it is the first FACS to be developed for a New World monkey species. One potential application of CalliFACS is to facilitate research into the evolution of communication and emotions in humans and other animals, since the common marmoset is an interesting intermediate model between rodents and other primates, and phylogenetically distant from humans, whilst sharing many neuro-anatomical [25, 38] and socio-ecological [28, 34, 82] characteristics. For example, in a recent application of FACS to compare AUs from two phylogenetically distantly related species (i.e. humans and dogs) when faced with specific emotionally-competent triggers [*sensu* 83], it was revealed that both species display completely different AUs, providing clues in how facial expressions evolved outside the primate group [84]. In more applied settings, such as when working with individuals in captivity (e.g. laboratories, zoos), CalliFACS is a useful tool to help improve welfare. Common marmosets have increased in popularity for research [39], particularly in the fields of neuroscience [25, 34, 38, 40, 85, 86], cognition [35, 87], and modelling of human diseases [40, 88–90]. As such, CalliFACS is an important tool, not only to aid in evaluating the welfare of common marmosets in experimental settings and before, during and after invasive procedures, but also to monitor species-specific behaviour, which must be taken into account to ensure unbiased experiments.

For the CalliFACS development, no direct muscle validation was performed (e.g. through electrical stimulation) due to: 1) technical and anatomical limitations, as a very small needle would be needed to deliver electric current into such small muscle fibres, as well as difficulty of detecting correct placements for the needles due to small facial features in the common marmoset face; and 2) ethical concerns, as individuals need to be anesthetised for a procedure that is classified as invasive. However, since 1) this procedure has been done in humans [5], chimpanzees [5] and rhesus macaques [6], 2) primate facial musculature is well conserved regarding its functional homologies [58], 3) a skeletal mimetic muscle always contracts in one way only, i.e. by getting shorter and pulling on its attachment (the skin), and finally, 4) several other AnimalFACS (e.g. [13, 14]) have been developed and applied successfully without this invasive procedure, there is now enough information available on facial muscle function validation in mammals. While it might be possible that facial muscles in common marmosets contract in different ways than we described in CalliFACS, due to the reasons stated above, this is highly unlikely.

Despite the many advantages of using FACS (e.g. objectivity, standardisation, high detail, allowing quantification of AUs and cross-species comparisons, see also [20]), its application is very time-consuming, both in terms of requiring coders to spend time studying FACS and obtaining certification for each species of interest, but also during the actual video-coding of facial movements, in which the frame-by-frame analysis of just a few minutes of video can take many hours or even days. Furthermore, due to the quick and subtle nature of facial expressions, FACS coding can only be used with video recordings of individuals, not in real time. Both factors, i.e. long time and asynchronous video analysis, may challenge the potential of CalliFACS as a welfare tool, as many settings may require an immediate evaluation of facial indicators of welfare. As such, one of the next research steps after developing CalliFACS will be to automate the detection of AUs (recently published in rhesus and long-tailed macaques: [91], and in horses: [preprint: 92]). The automation of AnimalFACS will not only decrease the amount of work, time and training needed for use, but also allow the development of software applications where AUs can easily and quickly be identified and their meaning potentially extracted. Likewise, the publication of CalliFACS will now open up the possibility for automatic detection of facial expressions in common marmosets in future studies.

## Supporting information

S1 Video **a**. AU1+2—Brow Raiser in normal speed and in slow motion, respectively. The presence of AU1+2 is indicated by an arrow. **b** AU1+2 is initiated during the head turn.
(ZIP)

S2 Video **a.** AU1+2—Brow Raiser in normal speed and in slow motion, respectively. The presence of AU1+2 is indicated by an arrow. **b** AU1+2 is initiated a couple of frames before the head up movement.
(ZIP)

S3 Video **a** and **b.** AU41—Glabella Lowerer in normal speed and in slow motion, respectively.
(ZIP)

S4 Video **a** and **b.** AU41—Glabella Lowerer in normal speed and in slow motion, respectively, during mastication.
(ZIP)

S5 Video **a** and **b.** AU41—Glabella Lowerer in normal speed and in slow motion, respectively, during mastication.
(ZIP)

S6 Video **a** and **b.** AU41—Glabella Lowerer in normal speed and in slow motion, respectively, during mastication.
(ZIP)

S7 Video **a** and **b.**AU6—Cheek Raiser in normal speed and in slow motion, respectively, during mastication.
(ZIP)

S8 Video **a** and **b.** AU6—Cheek Raiser in normal speed and in slow motion, respectively.
(ZIP)

S9 Video AU43—Eye closure. (ZIP)

S10 Video **a** and **b.** AU45—Blink in normal speed and in slow motion, respectively.
(ZIP)

S11 Video **a** and **b.** AU47—Half Blink in normal speed and in slow motion, respectively.
(ZIP)

S12 Video **a** and **b.** AU47—Half Blink in normal speed and in slow motion, respectively.
(ZIP)

S13 Video **a** and **b.** AU47—Half Blink in normal speed and in slow motion, respectively.
(ZIP)

S14 Video **a** and **b.** AU109+110—Nose Wrinkler + Upper Lip Raiser in normal speed and in slow motion, respectively.
(ZIP)

S15 Video AU109+110—Nose Wrinkler + Upper Lip Raiser. (ZIP)

S16 Video **a** and **b.** AU109+110—Nose Wrinkler + Upper Lip Raiser in normal speed and in slow motion, respectively.
(ZIP)

S17 Video **a** and **b.** AU110—Upper Lip Raiser in normal speed and in slow motion, respectively.
(ZIP)

S18 Video **a** and **b.** AU110—Upper Lip Raiser in normal speed and in slow motion, respectively.
(ZIP)

S19 Video **a** and **b.** AU110—Upper Lip Raiser in normal speed and in slow motion, respectively.
(ZIP)

S20 Video **a** and **b.** AU12—Lip Corner Puller in normal speed and in slow motion, respectively.
(ZIP)

S21 Video **a** and **b.** AU12—Lip Corner Puller in normal speed and in slow motion, respectively.
(ZIP)

S22 Video **a** and **b.** AU12—Lip Corner Puller in normal speed and in slow motion, respectively.
(ZIP)

S23 Video **a** and **b.** AU16—Lower Lip Depressor in normal speed and in slow motion, respectively.
(ZIP)

S24 Video **a** and **b.** AU16—Lower Lip Depressor in normal speed and in slow motion, respectively.
(ZIP)

S25 Video **a** and **b.** AU16—Lower Lip Depressor in normal speed and in slow motion, respectively. AU16 is present during the whole video, as the lower teeth are exposed for the duration of the video.
(ZIP)

S26 Video **a** and **b.** AU118—Lip Pucker in normal speed and in slow motion, respectively.
(ZIP)

S27 Video **a** and **b.** AU118—Lip Pucker in normal speed and in slow motion, respectively. AU118 is initiated during the tongue show (AD19). The last frames of the clip show the lip corners returning to neutral after AU118.
(ZIP)

S28 Video **a** and **b.** AU118—Lip Pucker in normal speed and in slow motion, respectively.
(ZIP)

S29 Video **a** and **b.** AU118—Lip Pucker in normal speed and in slow motion, respectively.
(ZIP)

S30 Video **a** and **b.** AU25—Lips Part in normal speed and in slow motion, respectively.
(ZIP)

S31 Video **a** and **b.** AU26—Jaw Drop in normal speed and in slow motion, respectively.
(ZIP)

S32 Video **a** and **b.** AU26—Jaw Drop in normal speed and in slow motion, respectively.
(ZIP)

S33 Video **a** and **b.** AU27—Mouth Stretch in normal speed and in slow motion, respectively.
(ZIP)

S34 Video **a** and **b.** AU27—Mouth Stretch in normal speed and in slow motion, respectively.
(ZIP)

S35 Video **a** and **b.** AU27—Mouth Stretch in the individual at the top of the video, in normal speed and in slow motion, respectively.
(ZIP)

S36 Video **a** and **b.** AU38—Nostril Dilator in normal speed and in slow motion, respectively.
(ZIP)

S37 Video **a** and **b.** AU38—Nostril Dilator in normal speed and in slow motion, respectively.
(ZIP)

S38 Video **a** and **b.** AD19—Tongue Show in normal speed and in slow motion, respectively.
(ZIP)

S39 Video **a** and **b.** AD190—Tongue Downwards in normal speed and in slow motion, respectively.
(ZIP)

S40 Video **a** and **b.** AD191—Tongue Curl in normal speed and in slow motion, respectively.
(ZIP)

S41 Video **a** and **b.** AD29—Jaw Thrust in normal speed and in slow motion, respectively.
(ZIP)

S42 Video **a** and **b.** AD29—Jaw Thrust in normal speed and in slow motion, respectively.
(ZIP)

S43 Video **a** and **b.** AD30—Jaw Sideways in normal speed and in slow motion, respectively.
(ZIP)

S44 Video **a** and **b.** AD30—Jaw Sideways in normal speed and in slow motion, respectively.
(ZIP)

S45 Video **a** and **b.** AD181—Lip Smacking in normal speed and in slow motion, respectively.
(ZIP)

S46 Video **a** and **b.** EAD1—Ears Forward in normal speed and in slow motion, respectively. Both individuals produce an EAD1 when turning the head forward and releasing it afterwards. In the top individual, only the EAD1 release is observed before the head turn, due to the tufts covering the ear.
(ZIP)

S47 Video **a** and **b.** EAD1—Ears Forward in normal speed and in slow motion, respectively. EAD1 is produced immediately after the release of EAD3, when the individual turns its head.
(ZIP)

S48 Video **a** and **b.** EAD3—Ears Flattener in normal speed and in slow motion, respectively.
(ZIP)

S49 Video **a** and **b.** EAD3—Ears Flattener in normal speed and in slow motion, respectively.
(ZIP)

S50 Video **a** and **b.** EAD105—Ears Downwards in normal speed and in slow motion, respectively.
(ZIP)

S51 Video **a** and **b.** EAD105—Ears Downwards in normal speed and in slow motion, respectively.
(ZIP)

S52 Video **a** and **b.** EAD3—Ears Flattener and EAD105—Ears Downwards in normal speed and in slow motion, respectively.
(ZIP)

S53 Video **a** and **b.** EAD3—Ears Flattener and EAD105—Ears Downwards in normal speed and in slow motion, respectively.
(ZIP)

S54 Video a and b. AD300—Tufts Upwards in normal speed and in slow motion, respectively.
(ZIP)

S55 Video **a** and **b.** AD300—Tufts Upwards in the individual at the bottom of the image, in normal speed and in slow motion, respectively.
(ZIP)

S56 Video **a** and **b.** AD301—Tufts Downwards in normal speed and in slow motion, respectively.
(ZIP)

S57 Video **a** and **b.** AD301—Tufts Downwards in normal speed and in slow motion, respectively.
(ZIP)

S58 Video **a** and **b.** AD301—Tufts Downwards in normal speed and in slow motion, respectively.
(ZIP)

S59 Video **a** and **b.** AD301—Tufts Downwards in normal speed and in slow motion, respectively.
(ZIP)

S60 Video **a** and **b.** AD301—Tufts Downwards in normal speed and in slow motion, respectively.
(ZIP)

S61 Video **a** and **b.** AD101—Scalp Retraction in normal speed and in slow motion, respectively.
(ZIP)

S62 Video **a** and **b.** AD101—Scalp Retraction in normal speed and in slow motion, respectively.
(ZIP)

S63 Video **a** and **b.** AD101—Scalp Retraction in normal speed and in slow motion, respectively.
(ZIP)

S64 Video **a** and **b.** AD101—Scalp Retraction in normal speed and in slow motion, respectively.
(ZIP)

S65 Video **a** and **b.** AD101—Scalp Retraction in normal speed and in slow motion, respectively. There is first a release of AD100 followed by another AD100.
(ZIP)

S66 Video AD101—Scalp Retraction. (ZIP)

S67 Video **a** and **b.** AD54—Head Down in normal speed and in slow motion, respectively.
(ZIP)

S68 Video AD55—Head Tilt Left in normal speed. (ZIP)

S69 Video **a** and **b.** AD56—Head Tilt Right in normal speed and in slow motion, respectively.
(ZIP)

S70 Video **a** and **b.** AD57—Head Forward in normal speed and in slow motion, respectively.
(ZIP)

S71 Video **a** and **b.** AD58—Head Back in individual on the left (after individual on the right jumps), in normal speed and in slow motion, respectively.
(ZIP)

S72 Video **a** and **b.** AD63—Eyes Up in normal speed and in slow motion, respectively.
(ZIP)

S73 Video **a** and **b.** AD80—Swallow in normal speed and in slow motion, respectively.
(ZIP)

S74 Video **a** and **b.** AD119—Lick in normal speed and in slow motion, respectively.
(ZIP)

S75 Video **a** and **b.** AD160—Body Shake in normal speed and in slow motion, respectively.
(ZIP)

S76 Video **a** and **b.** AD160—Body Shake in normal speed and in slow motion, respectively.
(ZIP)

S1 Text (DOCX)
