# Supplementary material for: CalliFACS: The common marmoset Facial Action Coding System
Source: PLoS One. 2022 May 17;17(5):e0266442. doi: 10.1371/journal.pone.0266442 (PMC9113598; doi:10.1371/journal.pone.0266442)
Supplement: S1 Text — (DOCX) [file pone.0266442.s077.docx]

**Supporting Information S1 Text for:**

**CalliFACS: The Common Marmoset Facial Action Coding System**

Catia Correia-Caeiro^1*^, Anne Burrows^2,3^, Duncan Andrew Wilson^1,4^, Abdelhady Abdelrahman^5^, Takako Miyabe-Nishiwaki^1^

^1^Primate Research Institute, Kyoto University, Inuyama, Japan

^2^Department of Physical Therapy, Duquesne University, Pittsburgh, Pennsylvania, USA

^3^Department of Anthropology, University of Pittsburgh, Pittsburgh, Pennsylvania, USA

^4^Graduate School of Letters, Kyoto University, Kyoto, Japan

^5^School of Health and Life Sciences, Glasgow Caledonian University, Glasgow, UK

***Corresponding author**

E-mail: catia_caeiro@hotmail.com (CCC)

**ORCID**: https://orcid.org/0000-0002-2819-6039

**Head and Eye Action Descriptors:**

Head movement in **marmosets** is more difficult to code than in **humans**, due to the hair surrounding the neck area. However, head movements recorded in video are possible to code based on the movement itself, or due to changes in the relative position of the head and body.

**AD51 - Head Turn Left:** The head moves to the left along a vertical axis.

**AD52 - Head Turn Right:** The head moves to the right along a vertical axis.

**AD53 - Head Up:** The head moves upwards.

**AD54 - Head Down:** The head moves downwards (**S67a** and **S67b** Videos).

**AD55 - Head Tilt Left:** The head rotates tilting to the left side (**S68** Video).

**AD56 - Head Tilt Right:** The head rotates tilting to the right side (**S69a** and **S69b** Videos).

**AD57 - Head Forward:** The head moves forward and away from the body. In a neutral position, the neck is not well-defined, but with AD57, the neck elongates forward and becomes visible (**S70a** and **S70b** Videos).

**AD58 - Head Back:** The head moves back and towards the body (**S71a** and **S71b** Videos).

In **humans**, eye movements are easy to detect as the coloured iris is surrounded by a large white sclera. In **marmosets**, eye movements can be more difficult to detect, since the eyes usually have a dark brown iris without any sclera visible in the neutral position. Marmoset eyes have a black round pupil that reacts to light, although in most lighting conditions the pupil is hard to distinguish from the iris. A small slit of white sclera can become visible on the outer eye corner when the eyeball moves laterally (**Fig 20**) aiding the detection of some eye movements. The nictitating membrane becomes more visible in the inner eye corner during some eye movements as well.

Head and eye positions tend to influence how each are perceived by humans (Otsuka & Clifford, 2018), so it is important to look for cues other than position alone, such as sclera visibility or clear movement.

**AD61 - Eyes Turn Left:** The eyes move to the left. In large movements the sclera may show on the right side of the eye (**Fig 20**).

**AD62 - Eyes Turn Right:** The eyes move to the right. In large movements the sclera may show on the left side of the eye.

**AD63 - Eyes Up:** The eyes move upwards (**S72a** and **S72b** Videos).

**AD64 - Eyes Down:** The eyes move downwards.


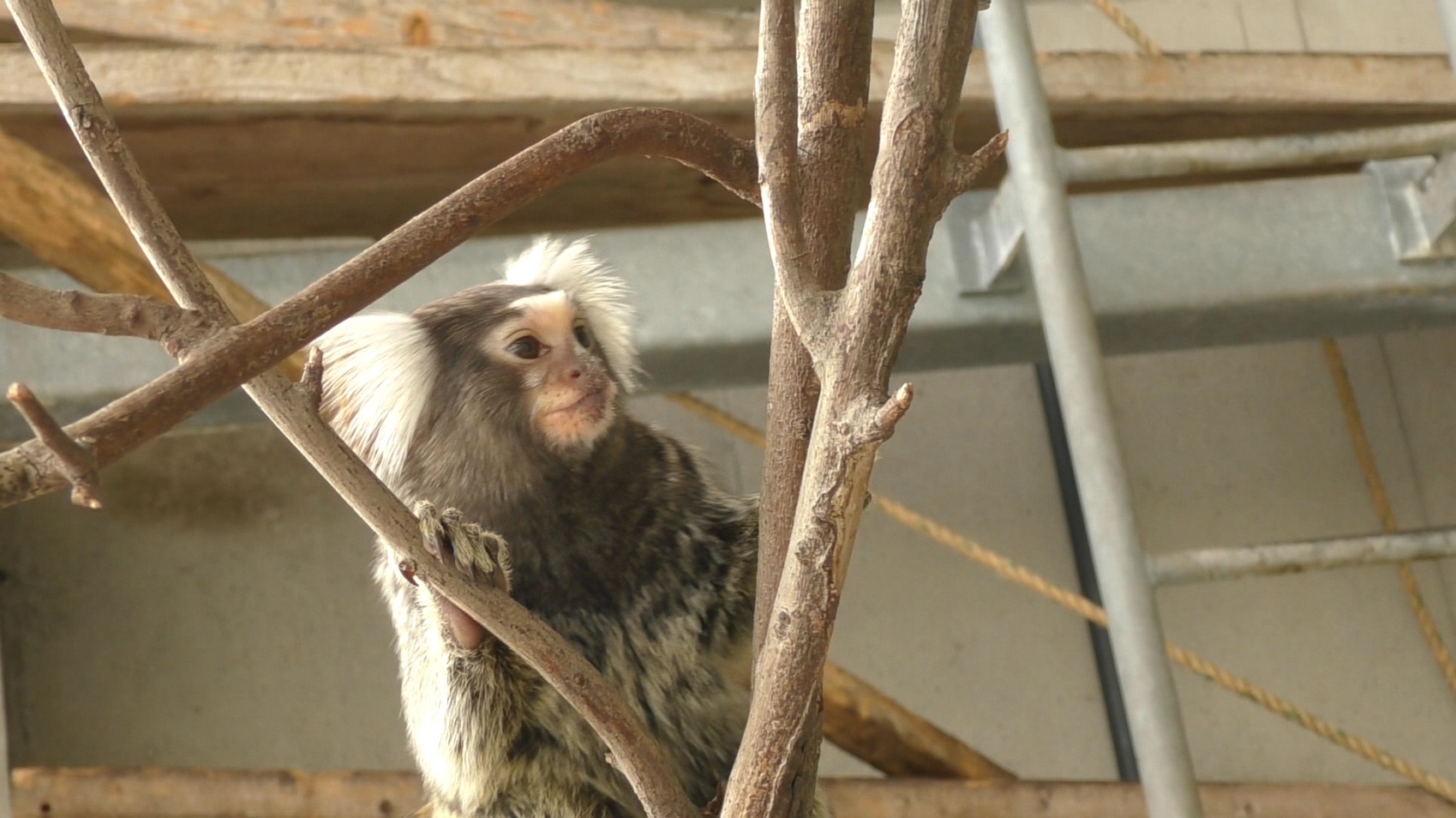

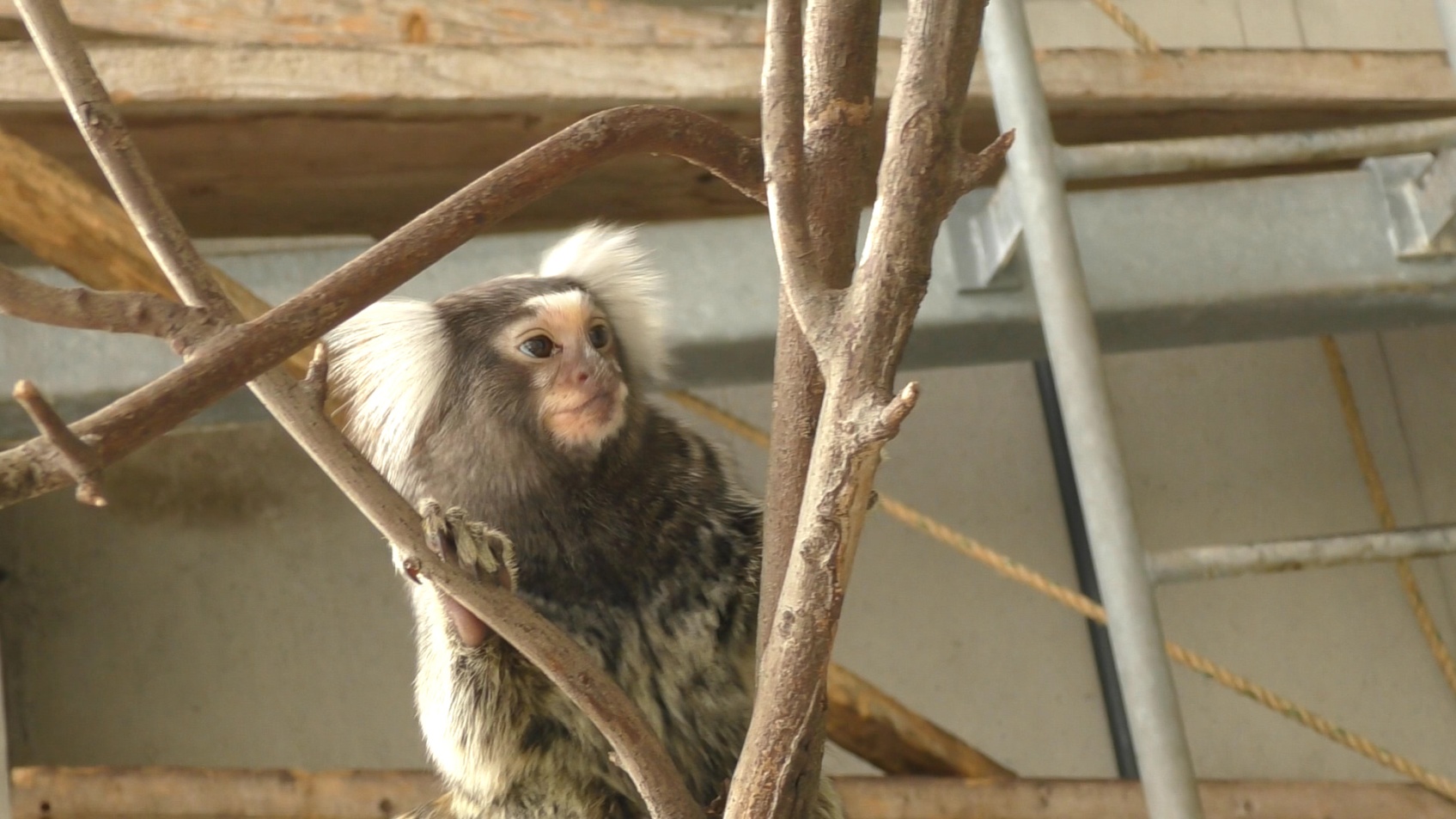


**Fig 20.** **AD61 - Eyes Turn Left.** Left: Neutral; Right: AD61 is present, with sclera exposure.

**Gross behaviour codes**

Gross behaviour codes are more general categories of behaviour that are not based on facial actions and may or may not involve the face. These codes are useful to score alongside AUs and other ADs as they can impact on or modify appearance changes.

**AD40 - Sniff:** This AD refers to the act of smelling, where global movement with varied intensity is observed around the nose and nostrils. Sniff was observed when inspecting objects (e.g. food), but also when not directed at any object or individual in particular. It is usually accompanied by AU38, although these two movements can also occur independently. AU38 may be difficult to detect depending on the angle and distance of the individual from the camera.

**AD50 - Vocalisations:** Marmosets display a wide range of vocalisations and most of them change the movement of the mouth and nose. This should be accounted for when coding facial behaviour.

**AD80 - Swallow:** Passing food, water or saliva from the mouth to the oesophagus through the action of a range of non-mimetic muscles. In marmosets, swallowing can produce movement on the fur on the ventral side of the neck and just below the neck (**S73a** and **S73b** Videos).

**AD81 - Chewing:** Feeding-related behaviour when food is introduced into the mouth and the jaws are moved repeatedly to break down the food, involves several AUs and ADs described above. However, AD81 should be coded alongside other AUs and ADs whenever mouth movements are primarily due to feeding-related behaviours.

**AD119 - Lick:** The individual licks something (e.g. object, person, self, conspecifics) and should be coded instead of AD19 or other tongue related ADs (**S74a** and **S74b** Videos).

**AD160 - Body Shake:** When the body and/or head is twisted, shaking the head and trunk skin, half-rotating the head and/or trunk quickly and repeatedly in a craniocaudal axis. Ear movements during or immediately following AD160 should not be coded as they are not the result of direct ear muscle action.

**Visibility codes**

**AD 70 - Frontal Region Not Visible:** When the brow and frontal region cannot be clearly seen and coded.

**AD 71 - Eyes Not Visible:** When the eyes cannot be clearly seen and coded.

**AD 72 - Lower Face Not Visible:** When the lower face cannot be clearly seen and coded.

**AD 73 - Entire Face Not Visible:** When the entire face is out of view and cannot be clearly seen and coded.

**AD74 - Ears Not Visible**: When the ears are out of view and cannot be coded.

# Glossary:

**Apex (of an AU)**: When the movement reaches the maximum intensity and no further increase in muscular action can be observed for that particular AU. The apex can be different each time the same AU is produced, both in how long it is maintained and regarding the maximum intensity.

**De-elongate**: The mouth (or other feature) appears to be shorter than usual in the horizontal plane.

**Dorsal plane**: Imaginary plane of reference that sections the trunk of an individual, parallel to the back and belly. It divides the body into dorsal (upper) and ventral (lower) segments.

**Elongate**: The mouth (or other feature) appears to be longer than usual in the horizontal plane. Opposite of de-elongate.

**External ear:** Portion of the ear external to the head, also designated as auricle, pinna, or concha.

**Facial morphology:** Series of complex facial traits, influenced by both genetics and environmental factors, unique to each individual, such as face and facial feature size, shape and location.

**Facial landmarks:** Key points or structures on the face which are common between individuals of a species. In FACS, they aid in identifying AUs.

**Facial nerve** (cranial nerve, CNVII): The seventh cranial nerve that supplies most of the muscles of the head, and in particular the ones involved in facial behaviour. The facial nerve leaves the lateral border of the corpus trapezoideum in the brain, near its cranial edge, between the fifth and eighth nerves, radiating to the face.

**False indicator**: When a cue used for identifying an AU is already present in the appearance on the neutral face, being thus dependent of the individual unique facial morphology.

**Flatten**: To decrease indentations or salient areas. For example, the lips may appear flattened against the teeth, i.e. they protrude less than usual.

**Glabella**: In humans, it is the space between the two eyebrows. In non-human animals, the glabella is located on the frontal region area, in the midline of the browridge.

**Holistic**: Same as gestalt or global. Refers to the whole that it is formed by several parts. In reference to facial expressions, it means encompassing the whole face/all facial features/all facial movements as one object (e.g. a sad facial expression is an holistic categorisation, while lip corners pulled downwards is one part of that facial expression). In reference to facial processing, it means the face is perceived globally as one single object, instead of by its features/parts.

**Homology (anatomical)**: Similarity between structures from two different species that diverged from a common ancestor. The function of these structures may or may not be identical.

**Mandible**: Lower jaw bone.

**Mastication**: Chewing.

**Mastication muscles**: muscles related to functions such as chewing, jaw movement, swallowing and vocalisations, that include the masseter, temporalis, pterygoid and digastric muscles. These muscles are not supplied by the facial nerve, except for the digastric muscle.

**Maxilla**: Upper jaw bone.

**Mental region**: Facial feature below the mouth, located in the same area as the human chin. The chin (mental protuberance in the skull bone) is a unique anatomical feature present only in humans, and thus in animals this area is referred to as the mental region.

**Mimetic muscles**: The muscles that are supplied by the facial nerve (cranial nerve, CNVII).

**Neutral** (in reference to an AU): Absence of any appearance change related to a particular AU, which can be observed before the onset and after the offset of an AU.

**Neutral face**: Absence of any appearance change in the face due to visible muscular contraction. It serves as a baseline comparison for coding the presence of AUs.

**Nictitating membrane**: Third eyelid more or less visible (depending on the species) in the inner eye corner. In some taxa (e.g. birds) this membrane slides across the eye for varied functions, but in mammals, including humans and marmosets tends to be immobile and undeveloped.

**Nostril wing or ala**: Portion of the nose around the nostril cavity on each side of the nose.

**Offset** (of an AU): Length of time from the end of the apex to the point where the muscle is no longer contracting (neutral).

**Onset** (of an AU): Length of time from the start of the movement till the apex.

**Pinna**: Projecting outer part of the ear that is visible.

**Protrude**: The lips (or other feature) move forward or out, away from the face, more than usual. Opposite of flatten.

**Rotation**: The movement of a part along its long axis. For example, the head rotates when looking left or right along its longer dimension (from top of the head till the neck).

**Stretch**: The lips (or other feature) are pulled and the skin stretched like a rubber band.

**Tighten**: The lips (or other feature) appear tight, the lips are not relaxed or loose. The muscle within the lips has contracted.

**Transverse plane:** Imaginary plane of reference that is perpendicular to the dorsal plane. It sections the body into cranial (front) and caudal (back) segments.

**Vibrissae**: Type of mammalian hair with tactile functions and dedicated nerve supply.
